# Developmental Validation of a MPS Workflow with a PCR-Based Short Amplicon Whole Mitochondrial Genome Panel

**DOI:** 10.3390/genes11111345

**Published:** 2020-11-13

**Authors:** Jennifer Churchill Cihlar, Christina Amory, Robert Lagacé, Chantal Roth, Walther Parson, Bruce Budowle

**Affiliations:** 1 Center for Human Identification, University of North Texas Health Science Center, 3500 Camp Bowie Boulevard, Fort Worth, TX 76107, USA; Bruce.Budowle@unthsc.edu; 2 Department of Microbiology, Immunology and Genetics, University of North Texas Health Science Center, 3500 Camp Bowie Boulevard, Fort Worth, TX 76107, USA; 3 Institute of Legal Medicine, Medical University of Innsbruck, 6020 Innsbruck, Austria; christina.amory@i-med.ac.at (C.A.); walther.parson@i-med.ac.at (W.P.); 4 Human Identification Group, Thermo Fisher Scientific, South San Francisco, CA 94080, USA; Robert.Lagace@thermofisher.com (R.L.); chantal.roth@thermofisher.com (C.R.); 5 Forensic Science Program, The Pennsylvania State University, University Park, PA 16802, USA

**Keywords:** mitochondrial DNA, massively parallel sequencing, Ion Torrent, developmental validation, SWGDAM guidelines

## Abstract

For the adoption of massively parallel sequencing (MPS) systems by forensic laboratories, validation studies on specific workflows are needed to support the feasibility of implementation and the reliability of the data they produce. As such, the whole mitochondrial genome sequencing methodology—Precision ID mtDNA Whole Genome Panel, Ion Chef, Ion S5, and Converge—has been subjected to a variety of developmental validation studies. These validation studies were completed in accordance with the Scientific Working Group on DNA Analysis Methods (SWGDAM) validation guidelines and assessed reproducibility, repeatability, accuracy, sensitivity, specificity to human DNA, and ability to analyze challenging (e.g., mixed, degraded, or low quantity) samples. Intra- and inter-run replicates produced an average maximum pairwise difference in variant frequency of 1.2%. Concordance with data generated with traditional Sanger sequencing and an orthogonal MPS platform methodology was used to assess accuracy, and generation of complete and concordant haplotypes at DNA input levels as low as 37.5 pg of nuclear DNA or 187.5 mitochondrial genome copies illustrated the sensitivity of the system. Overall, data presented herein demonstrate that highly accurate and reproducible results were generated for a variety of sample qualities and quantities, supporting the reliability of this specific whole genome mitochondrial DNA MPS system for analysis of forensic biological evidence.

## 1. Introduction

The mitochondrial genome (mtGenome) has a number of characteristics that make it useful for analyzing forensic biological evidence. With a higher copy number per cell compared to the nuclear genome, mitochondrial DNA (mtDNA) is often analyzed in evidence where there is little to no nuclear DNA. In addition, the mtGenome’s uniparental inheritance and lack of recombination make this genetic marker particularly informative for cases involving maternal lineage analyses. With the availability of databases such as EMPOP [1] and PhyloTree [2], the mtGenome’s well-characterized phylogeny also offers access to phylogenetic information, which can support quality control of data and various population studies. As such, mtDNA analysis has been used in forensic examinations and investigations for over two decades [3,4,5,6,7,8].

Mitochondrial DNA testing in forensic laboratories has traditionally been performed with capillary electrophoresis (CE)-based Sanger sequencing. Due to the workflow’s high cost and the labor demands involved in sequencing the entire mtGenome with this technology, laboratories generally have targeted the highly polymorphic control region. The CE-based Sanger sequencing system, although limited in its ability to sequence large expanses of the mtGenome on a practical level, has been demonstrated to be highly reliable. However, with the development and maturation of massively parallel sequencing (MPS) technologies [9,10,11,12,13,14,15,16,17,18,19], there is interest in enhancing mtDNA analysis, with some laboratories already tackling the validation and implementation process [20,21,22,23,24]. Analysis of the mtGenome offers a good first step to transition MPS workflows as current CE-based workflows are already sequence-based, making data analysis of MPS results a more familiar and similar process. MPS makes it feasible to sequence the entire mtGenome with less labor, less sample consumption, and an overall lower cost per nucleotide. Furthermore, MPS provides an increase in sensitivity over currently-used CE-based technologies allowing for detection and characterization of lower-level heteroplasmies [20,24,25,26]. Additionally, the increase in information, provided by evaluating the entire mtGenome, offers the potential for an increase in discrimination power and phylogenetic resolution [10,26,27,28]. As databases with whole mtGenome data continue to grow [10,14,24,26,27,29,30,31,32], some laboratories are opting to employ multiplexes that target the entire mtGenome [21,24].

Due to the often-degraded nature of the forensic evidence samples, multiplex development has focused on small amplicons that span the mtGenome [11,12,20,24,33,34,35]. The Precision ID mtDNA Whole Genome Panel (Thermo Fisher Scientific, Waltham, MD, USA) is a multiplex designed to amplify the entire mitochondrial genome in a tiled, overlapping manner. This multiplex is composed of two primer pools with 81 primer pairs each, generating amplicons with an average size of 163 base-pairs (bps) and an average amplicon overlap of 11 bps [36]. Each primer pool also contains degenerate primers, 119 and 164 degenerate primers in pool one and pool two, respectively, to accommodate known SNPs residing in primer-binding sites to minimize amplicon dropout [24,36]. These potential primer-binding SNP sites were bioinformatically identified using the 2014 phase 3 1000 Genomes Project (variant frequency > 5%), and 2014 MITOMAP (variant frequency > 700 count) databases [24,36,37,38,39]. The control region primers were designed using EMPOP (2013) [1] data to avoid high frequency SNPs and indels if possible. For primers where it was unavoidable, a degenerate primer was included for any SNP residing within a primer binding site with an EMPOP frequency of 2% or more. This Precision ID mtDNA Whole Genome Panel was designed for use on a platform consisting of the Ion Chef (Thermo Fisher Scientific) and Ion S5 sequencer (Thermo Fisher Scientific). This MPS system has already been well described in the literature and shows promise for forensic applications [15,24,26,28,30,31,40,41,42,43,44,45].

Developmental validation studies for the mtGenome workflow consisting of the Precision ID mtDNA Whole Genome Panel, Ion Chef, and Ion S5, combined with Converge software (Thermo Fisher Scientific) for data analysis were performed and are detailed herein. Validation studies were completed in accordance with the Scientific Working Group on DNA Analysis Methods (SWGDAM) Validation Guidelines for Forensic DNA Analysis Methods, which is intended to serve as a guide for forensic laboratories in designing validation studies consistent with the Federal Bureau of Investigation (FBI) Director’s Quality Assurance Standards (QAS) [46]. Data generated by this workflow were compared to traditionally prepared Sanger sequencing data and MPS data on an orthogonal platform for accuracy and concordance. Each of these studies provide data that support the validity and reliability of the Precision ID mtDNA Whole Genome Panel, Ion Chef, Ion S5, and Converge workflow for forensic applications.

## 2. Materials and Methods

### 2.1. Previous Data

Strobl et al. [24] detailed sequencing results of 521 mtGenomes from 24 populations, which were sequenced as part 1 of the developmental validation designed to evaluate the performance of the Precision ID mtDNA Whole Genome Panel and supporting platform. These mtGenomes covered all major clades of the mitochondrial phylogeny (except O and S), allowing for the effect of sequence motifs specific to phylogenetic backgrounds on primer-binding and amplification efficiency to be evaluated.

The sequencing and analytical methods used for the analyses of these 521 samples are described in Strobl et al. [24]. Briefly, after amplifying the mtGenome with the Precision ID mtDNA Whole Genome Panel, libraries were manually prepared and then templated on the Ion Chef (Thermo Fisher Scientific). Templated ion sphere particles (ISPs) were sequenced on the Ion S5 (Thermo Fisher Scientific). Primary data analyses were performed with the Torrent Suite software followed by manual curation by two independent scientists using Integrative Genomics Viewer (IGV) [47,48]. Data in this study also were analyzed with Converge software (Thermo Fisher Scientific).

### 2.2. Samples and Studies

A total of 686 samples (including positive and negative controls) were sequenced in a series of studies designed to evaluate the performance of the Precision ID mtDNA Whole Genome Panel, Ion Chef, Ion S5, and Converge workflow (Table S1). These studies were completed at both the Institute of Legal Medicine (GMI), Medical University of Innsbruck, Austria and the University of North Texas Health Science Center’s Center for Human Identification (UNTCHI), in Fort Worth, Texas, USA. Collection and use of human samples for these studies were approved by the Institutional Review Board for the University of North Texas Health Science Center in Fort Worth, TX. The DNA extraction procedures for the samples included in these studies are listed in Table S1. Both Quantifiler Trio [49] and an in-house mtDNA qPCR assay [50] were used for quantification of nuclear and/or mtDNA in the samples sequenced herein.

### 2.3. Concordance Data

Previously generated Sanger sequencing and MPS data on samples sequenced in the accuracy and mock casework studies were used for concordance evaluations. The Sanger data from GMI were generated with the workflow and guidelines detailed in Parson et al. [8,51,52]. The Sanger data from UNTCHI were generated with the workflow detailed in Davis et al. [11]. MPS data, previously generated on the MiSeq (Illumina, San Diego, CA, USA) at UNTCHI, are described in King et al. [10].

### 2.4. Library Preparation

Manual and automated library preparation methods were used in this study. For both methodologies, the mtGenome of each sample was amplified with the Precision ID mtDNA Whole Genome Panel following the manufacturer’s recommended protocols [36]. DNA input amounts varied based on the specific validation study and are described in Table S1. Manual libraries were prepared using the Precision ID Library Kit (Thermo Fisher Scientific) following the manufacturer’s recommended protocols [36] for the “2-in-1 method”. Automated libraries were prepared on the Ion Chef with the Precision ID DL8 Kit (Thermo Fisher Scientific) following the manufacturer’s recommended protocols [36].

### 2.5. Templating and Sequencing

Each library was quantified using the Ion Library TaqMan Quantitation Kit (Thermo Fisher Scientific) and normalized to 30 pM when possible (libraries with less than 30 pM were used neat). Details on how libraries were pooled for each sequencing run are listed in Table S1. Template preparation was completed on the Ion Chef following the manufacturer’s recommended protocols [36]. Once the ion chip was loaded with templated ISPs, the chip was placed on an Ion S5 sequencer for sequencing. The Ion S5 Precision ID Chef and Sequencing Kit, Ion S5, and the manufacturer’s recommended protocols [36] were used for sequencing.

### 2.6. Data Analysis

Primary data analyses were performed with the Torrent Suite software (version details included in Table S2). A revised Cambridge Reference Sequence (rCRS) reference genome [53] with nucleotides 1–80 repeated after position 16,569 was used to allow for accurate alignment. Alignment and variant calling were performed with the HIDGenotyper v2.1 plugin and Converge software v2.1 [54]. Sequence variant frequencies indicated the read depth of a particular nucleotide (or indel) relative to the total read depth at that nucleotide position (e.g., A: 10 reads (X); G: 90X; 10/100 = 10% sequence variant frequency). Sequence variant frequency thresholds of 10% for point heteroplasmies, 30% for insertions, and 40% for deletions were used for this study and were chosen based on preliminary assessment of the data described herein. For the insertion threshold, the total number of reads indicating the presence of an insertion relative to the total read depth at that nucleotide position was used to indicate an insertion was present for variant calling. The dominant variant was used to indicate which nucleotide(s) was inserted when generating haplotype calls. The frequency of the dominant variant indicated the number of reads with the dominant variant relative to the total number of reads indicating the presence of an insertion. A minimum read depth threshold of 20X for sequence variant calling was also used in this study to minimize the inclusion of exogenous DNA or noise as true mtDNA variation. The remaining editable parameters used in this study for the HIDGenotyper plugin are included in Table S3. After automated variant calling with Converge, manual verification of each sequence variant was performed using the IGV instance of Converge [47,48,54]. Sequence variants not labeled as “confirmed” were flagged for review by an analyst, which was performed during this manual verification process for the haplotypes generated in this study. Converge also provides a button linking the user to the EMPOP website [1] and a method for exporting an EMPOP compatible haplotype. These features were used to confirm nomenclature and haplotype calls in EMPOP [1,29]. Median read depth values for amplicons were calculated by Converge. Amplicon balance was calculated by comparing the median read depth of an amplicon to the median read depth of all amplicons for that sample (e.g., 100X/150X = 0.67), where 1.0 indicates balanced amplicons. Strand bias calculations compared the number of reads in one direction to the number of reads in the other direction at a particular nucleotide position (e.g., 1 − (read direction with the smaller number of reads/read direction with the larger number of reads); 1 − (5X/50X) = 0.9), where 0 indicated no strand bias and 1.0 indicated the presence of reads in only one direction. Sequencing run metrics were taken from the Run Summary PDF in the Torrent Browser. The relative read depth for negative controls was calculated as the read depth of the negative control compared to the read depth of a positive control in the same sequencing run for each nucleotide position (e.g., (50X/2000X) × 100 = 2.5%). Data handling and statistical analyses were performed with Excel (Microsoft Corporation, Redmond, WA, USA) and R v3.3.2 and RStudio v1.0.136 (R Core Team, 2016).

## 3. Results and Discussion

A developmental validation study is intended to generate data that aid in determining the conditions and limitations of a new methodology for use in forensic laboratories. As such, the thresholds set for analysis in this study were used to illustrate performance of the overall workflow and are not intended to be used as universal thresholds. Results are presented from a series of validation studies performed in accordance with SWGDAM Validation Guidelines for Forensic DNA Analysis Methods [46] to assess the Precision ID mtDNA Whole Genome Panel, Ion Chef, Ion S5, and Converge workflow’s reproducibility, repeatability, accuracy, sensitivity, specificity to human DNA, and overall ability to analyze challenged samples. An overall assessment of the workflow’s efficacy and reliability for mtDNA analysis is described below.

### 3.1. Sequencing Run Performance

Sequencing run metrics help assess the performance of the run and data quality generated by the run before progressing to mtGenome data analysis. The amount of raw data generated, the quality of the generated libraries, or issues that arise during the library preparation or sequencing process can indicate if the results are of sufficient quality to proceed. Run metrics, including the ISP loading, median read length, final library ISPs, etc., for each of the 33 sequencing runs included in this developmental validation are provided in Table S2.

If a run occasionally fell outside of the performance metrics’ target ranges [36], the run may have still produced sufficient data for analysis and interpretation. However, performance metric values that deviated substantially from the targeted range could negatively impact other quality metrics and decrease the quantity of data produced from the run. A total of 32 of the 33 (97%) sequencing runs performed similarly. However, run 12 resulted in only 198,416 (1.2%) usable reads for data analysis (Table S2). Due to the performance of run 12, this run was removed from subsequent analyses in this study.

For the workflow described herein, the PCR products and libraries were not completely consumed during the workflow. Therefore, when low-performing runs were obtained, such as the one described above, PCR products and libraries remained for re-sequencing, without having to consume any additional sample DNA.

### 3.2. Reproducibility and Repeatability

The precision of the Precision ID mtDNA Whole Genome Panel, Ion Chef, Ion S5, and Converge workflow was assessed with data from the eight reproducibility and repeatability runs (runs 1–8; Table S1) completed in this study, where the same samples were repeatedly typed by two different laboratories. DNAs from two sets of samples were used to prepare libraries twice, and these libraries then were sequenced twice on two separate chips. This process generated eight sequencing runs of data designed to capture variability between library preparations, sequencing runs, and laboratories.

**Figure 1:**
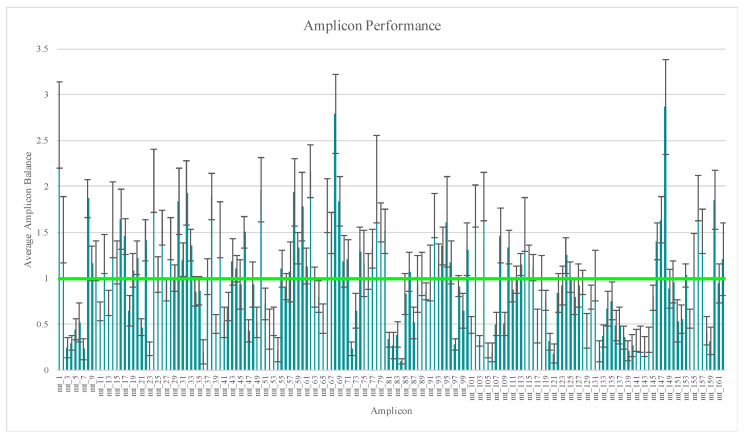
Average amplicon balance for the 112 samples included in the reproducibility and repeatability study. Vertical bars represent one standard deviation. Horizontal bar represents balanced amplicons.

A total of eight replicates were generated from each of the 14 distinct samples (*n* = 112) evaluated in this part of the study. Two replicates of sample RR2 yielded regions of the mtGenome below the 20X read depth threshold used for variant calling (Table S3). However, 99.4% and 98.8% of these mtGenomes reached or exceeded the 20X threshold. This region of lower read depth in RR2 likely is due to a decrease in amplification efficiency caused by a SNP in the primer-binding region of amplicon mt_52 at nucleotide position 5262. The effect of SNPs in primer-binding sites on data analysis with this workflow was discussed previously by Strobl et al. [24] and Cihlar et al. [55]. Average amplicon balance was calculated across these 112 samples and ranged from 0.10 (±0.03) for amplicon mt_84 to 2.87 (±0.52) for amplicon mt_148 (Figure 1), with similar high and low performing amplicons as those reported in previous studies [24,26,30,41,44]. A more in-depth look at amplicon performance, a comparison of the two primer pools’ performance, and a discussion regarding library pooling strategies can be found in part one of this developmental validation study [24].

The haplotypes from the 14 distinct samples included in the reproducibility and repeatability study are listed in Table S4. Data for the 007 control DNA were consistent with data reported previously by Cihlar et al. [41], with the exception of the 309del variant called in this study. Despite the use of similar thresholds and workflows, 309del was not called in both studies, and one source of variation between the two studies may be due to use of different lots of the 007 control DNA [41]. Data for the 9947A control DNA also were consistent with data reported previously in Cihlar et al. [41], with the exception of the dominant variant for the insertion at nucleotide position 309. For each replicate, 309.1C was the dominant variant, with 58.2% (±7.6%) on average of the insertion reads. The 309.2C reads were present at 34.8% (±5.6%) on average of the insertion reads. The platform’s difficulty in sequencing through homopolymeric regions and use of different lots of the control DNA likely are sources of variation between these two studies [41]. After a manual review of the data, the haplotype calls across all eight replicates were concordant. However, 10 sequence variants (1.2% of the total), while present in the data, did not reach the sequence variant frequency thresholds set for analysis in this study. In samples 007 and RR8, the dominant variant present for the 573.1 insertion (i.e., number of nucleotides present in the homopolymer) varied in two out of the eight replicates. A point heteroplasmy (11562R in RR1 and 12501R in RR8) fell just below the 10% threshold in one of the eight replicates for each sample. The 16192del in sample RR11 reached the 40% threshold in only four of the eight replicates sequenced. Finally, 309.1C did not reach the 30% threshold in each of the replicates for samples RR1, RR2, RR3 RR4, and RR8. Additionally, the 309.1C sequence variant was not called by Converge in two of the eight replicates for 9947A despite sequence variant frequencies of 73.1% and 74.8% due to the strand bias threshold set in Converge. Strand bias helps identify when an equivalent number of reads are generated from sequencing the targeted amplicon in both directions and is an additional threshold to consider for analysis. With a strand bias of 0.97, the reads spanning this region for 9947A were largely in one direction; yet the correct variant call was made manually. Finally, the 248R point heteroplasmy in samples RR5 and RR7 was called by Converge in half the replicates for each sample despite manual review of the data indicating the point heteroplasmy was present in all replicates. Both of these samples also had a deletion at nucleotide position 249. This deletion lies in the small overlap of amplicons mt_2 and mt_3, often making alignment difficult (Figure S1). Manual review of the data readily identified the variant and highlights a region that might require visual inspection by an analyst with current software tools. The 309del variant was called initially in four of the 14 (28.6%) reproducibility and repeatability samples using the thresholds set for analysis in this study. However, based on the concordance data and EMPOP data [1], which indicate that 309del is a rare variant, these 309del calls were considered false deletions and not included in the final haplotype calls in Table S4.

**Figure 2:**
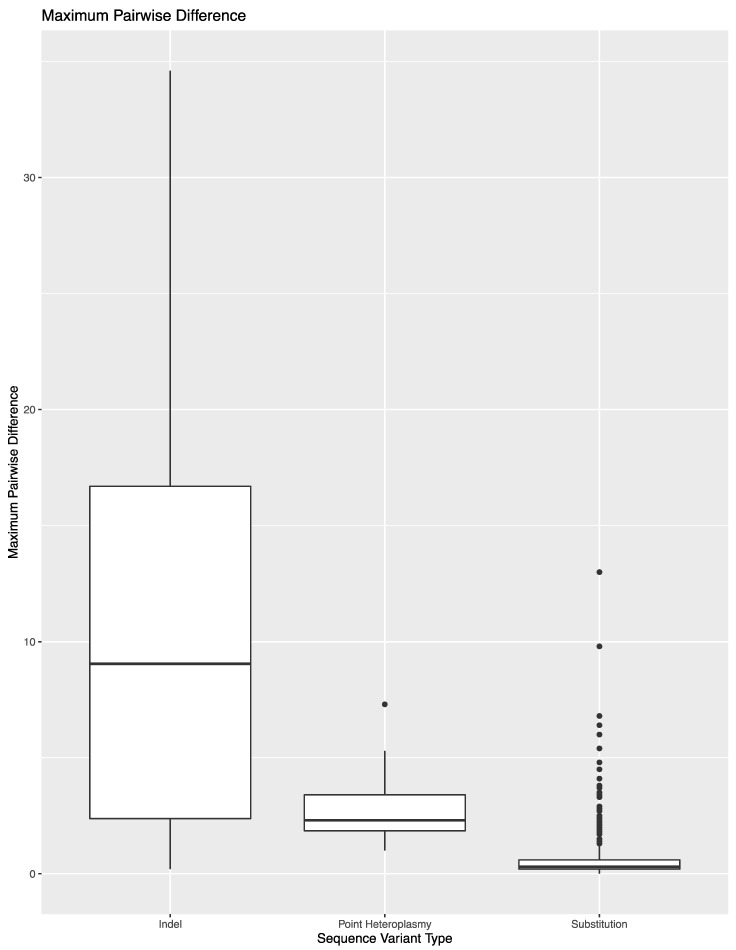
Maximum pairwise difference in variant frequency across the 850 sequence variants in the reproducibility and repeatability study grouped by sequence variant type.

**Figure 3:**
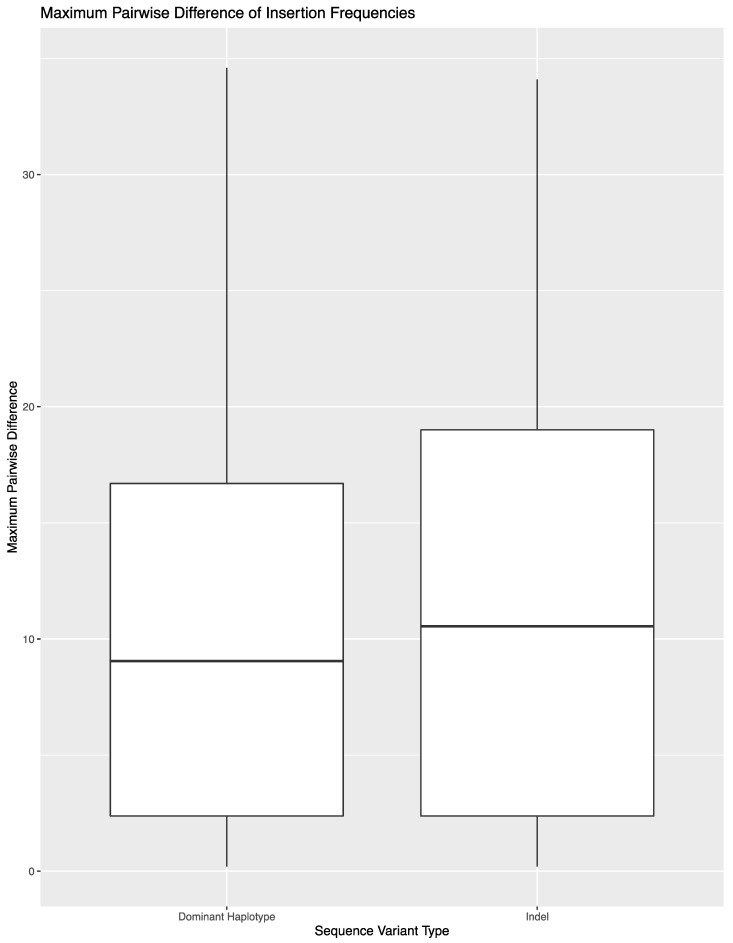
Maximum pairwise difference in variant frequency across the 52 indels in the reproducibility and repeatability study grouped by indel variant frequency as a whole or the frequency of the specific dominant haplotype.

To expand on the extent of variability across library preparations, sequencing runs, or laboratories, the variant frequency of the 850 sequence variants in these 14 samples was used to calculate the maximum pairwise difference in variant frequency across the eight replicates per sample (Figure 2). The average maximum pairwise difference in variant frequency across all 850 sequence variants was 1.2% (±3.4%). However, the average and spread of this metric varied by type of sequence variant. For substitutions, the average maximum pairwise difference in variant frequency was 0.6% (±0.9%). The outliers shown in Figure 2 correspond to substitutions that lead to the formation of longer homopolymers (e.g., 460C and 16189C). Difficulties in determining the exact number of nucleotides present in homopolymers lend to these sequence variants performing more similar to indels when calculating maximum pairwise difference. A total of 690 of the 783 substitutions (88.1%) had a maximum pairwise difference of less than 1%, with an average of 0.4 (±0.2%). The point heteroplasmies had an average maximum pairwise difference of 2.9% (±1.7%). The outlier in Figure 2 for point heteroplasmies is 248R, which was discussed above. The indels had an average maximum pairwise difference of 11.6 (±9.4). Again, the higher average and greater spread were expected for indels due to the platform’s difficulty in sequencing through and determining the exact number of nucleotides present in homopolymeric regions. This trend is reflected in the higher thresholds used for calling insertions and deletions in this study. The frequency of the dominant variant (i.e., the number of reads with the most prevalent haplotype/insertion relative to the number of reads reflecting the presence of an insertion) was also used to calculate the maximum pairwise difference for indels (Figure 3). The decrease in average (10.7% (±8.7%)) reflects the presence of reads with a variable number of nucleotides present for indels.

Overall, these data reflect the generation of highly reproducible haplotype calls. Due to the quantitative nature of MPS data, situations may be encountered where variants may be present but lie just below the thresholds that have been set for analysis. Laboratories will have to consider the possible variability in sequence variant frequencies between library preparations, sequencing runs, laboratories, and lot numbers of cell line DNAs when evaluating point heteroplasmies and indels.

### 3.3. Contamination

The contamination study was performed to help identify the level and potential sources of any background signal present in negative controls. This evaluation of the background signal includes an assessment of the potential or level of amplification of exogenous DNA, which could originate from the reagents, consumables, equipment used during the workflow, the operator completing the work, and the laboratory environment. Runs 9, 10, and 11 (Table S1; run 12 was dropped from analysis as described above) were designed to detect any run-to-run carry-over. Each of these runs included 16 samples in which one automated library preparation run was carried-out on the Ion Chef and followed by a second library preparation run on the Ion Chef with a checkerboard pattern of samples and negative controls. These sequencing runs included samples with DNA input amounts of 1 ng or 10 ng, which are values well over the manufacturer’s recommended input of DNA in an effort to exacerbate the potential for carry-over. Additionally, the negative controls in this study, 70 in total including those from runs 9, 10, and 11, were included in the contamination assessment.

**Figure 4:**
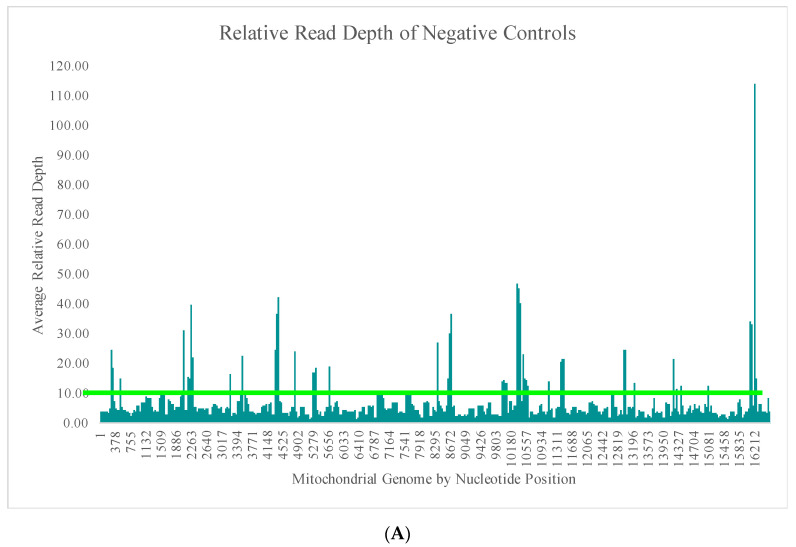

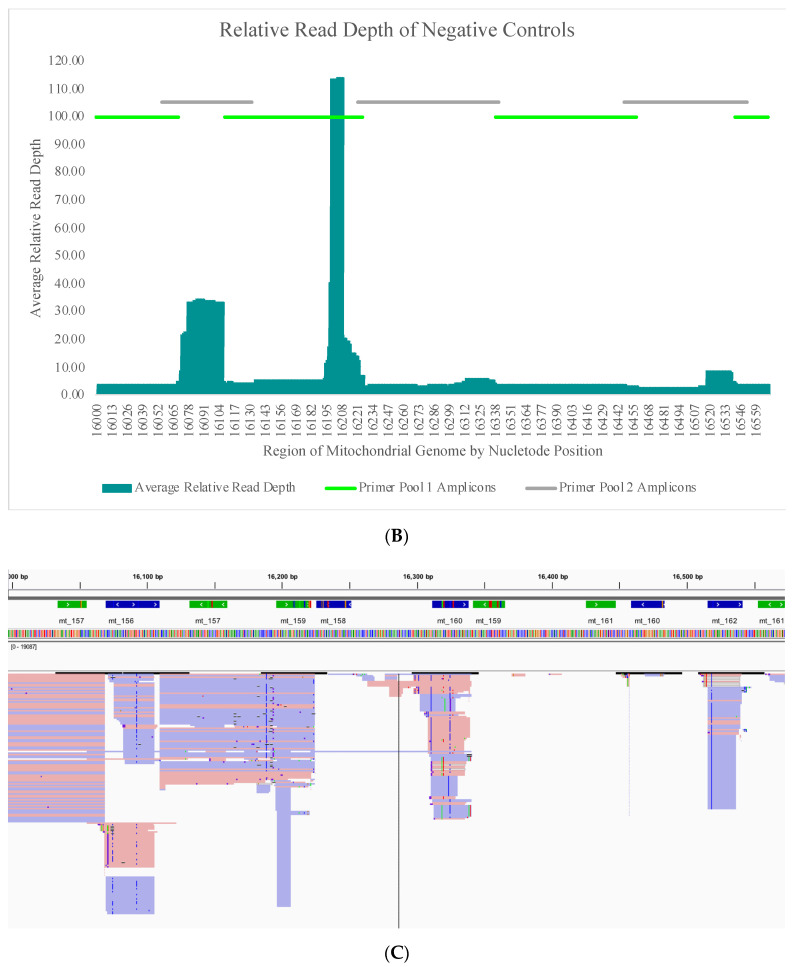

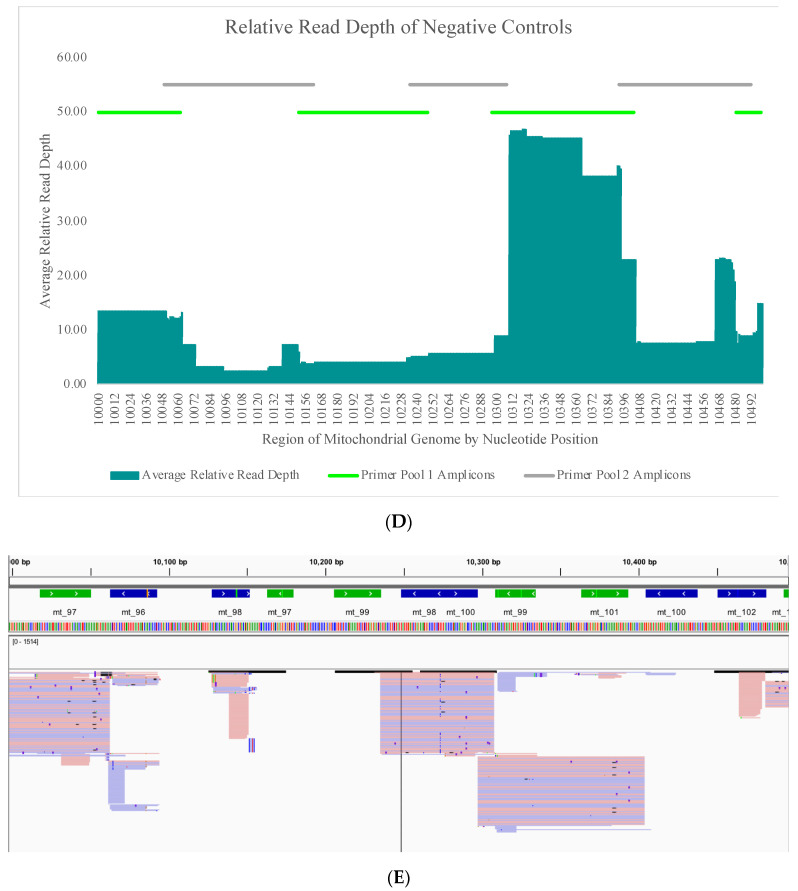
(**A**) The average relative read depth across the mtGenome for the 70 negative controls included in this study. The horizontal bar illustrates the 10% threshold. (**B**) The average relative read depth across a region of the mtGenome for the 70 negative controls included in this study. The horizontal bars indicate the location of targeted amplicons, and the two colors show the two primer pools included in the multiplex. (**C**) Screen shot from Integrative Genomics Viewer (IGV) of the reads of one negative control mapped to the same region illustrated in (**B**). (**D**) The average relative read depth across a region of the mtGenome for the 70 negative controls included in this study. The horizontal bars indicate the location of targeted amplicons, and the two colors show the two primer pools included in the multiplex. (**E**) Screen shot from IGV of the reads of one negative control mapped to the same region illustrated in (**D**).

The average read depth of these 70 negative controls ranged from 13.1X (±24.5X) to 689.7X (±2315.7X) across the mtGenome. The average read depth was below 200X for 84.8% of the mtGenome. Due to the quantitative nature of MPS data and the thresholds used for data analysis, the relative read depth (i.e., the read depth of the negative control compared to the read depth of a positive control in the same run for each nucleotide position) was evaluated. The average relative read depth for the 70 negative controls ranged from 1.3% (±1.9%) to 113.9% (±555.3%) across the mtGenome (Figure 4A). The average relative read depth was below 10% for 93.9% of the mtGenome. The remainder of the mtGenome rose above this 10% threshold in amplicons that were smaller than the 163 bp average size of the targeted amplicons generated by this panel suggesting the majority of these reads did not span their respective amplicons. Figure 4B–E illustrate the likely origin of these reads by focusing in on two regions containing the highest read depth (nucleotides 16,000 through 16,569 and nucleotides 10,000 through 10,500). These short reads, compared with the locations of targeted amplicons (horizontal bars in Figure 4B,D), are likely unused primer, short alternative PCR products generated by mismatched amplicons, or the overlapping regions of targeted amplicons.

#### Bioinformatic Negatives

During library preparation, barcodes (i.e., short sequences of nucleotides) are ligated to each PCR product to identify which sample that PCR product was amplified from during the analytical process. These barcode sequences allow for an increase in throughput, but barcode sequences also introduce another potential source of contamination. Thus, data from 32 bioinformatic negatives were evaluated as part of the contamination study. This assessment involved the analysis of data from 16 barcodes in runs 9 and 10 (Table S1), which included samples with 1 ng and 10 ng of input DNA, respectively, (both well-over the manufacturer recommended amount of input DNA), which were not associated with any sample during the library preparation process. This analysis offered the opportunity to detect potential exogenous barcode contamination, carry-over from previous runs, or demultiplexing errors.

**Figure 5:**
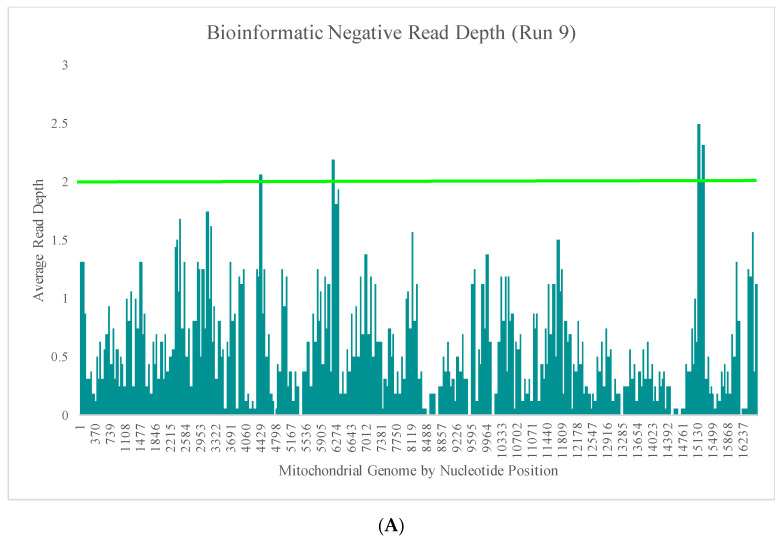

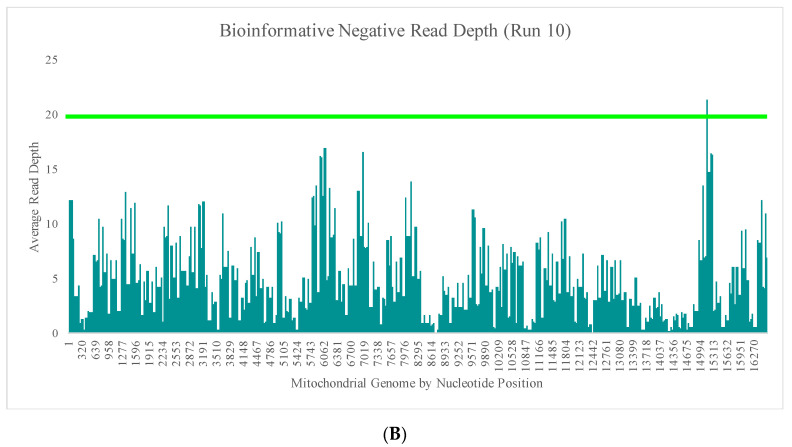
(**A**) The average read depth across the mtGenome for the 16 bioinformatic negatives included in run 9 (1 ng input DNA). The horizontal bar indicates an average read depth of 2X. (**B**) The average read depth across the mtGenome for the 16 bioinformatic negatives included in run 10 (10 ng input DNA). The horizontal bar indicates an average read depth of 20X.

Runs 9 and 10 were analyzed separately due to the differences in DNA input values of the positive samples included in the sequencing runs. The average read depth for the bioinformatic negatives in run 9 ranged from 0X (±0X) to 2.5X (±2.8X) across the mtGenome (Figure 5A). The average read depth for these 16 bioinformatic negatives was less than 2X for 99.1% of the nucleotide positions. The regions of the mtGenome where the average read depth reached 2X or greater were the small areas of overlap between amplicons (Figure 5A). The average read depth for the bioinformatic negatives in run 10 ranged from 0X (±0X) to 21.4X (±17.9) across the mtGenome (Figure 5B). The average read depth for these 16 bioinformatic negatives was less than 20X for 99.9% of the nucleotide positions and less than 10X for 96.4% of the nucleotide positions. The regions of the mtGenome where the average read depth reached 20X or greater were, again, the small areas of overlap between amplicons (Figure 5B).

This contamination assessment provides insight into how to critically evaluate data from negative controls in MPS runs and helps determine the origin of reads observed in negative controls. Updates to the Converge software (v2.2) address removal of the previously mentioned short reads for a better representation of amplification of targeted amplicons in exogenous DNA (Figure S2) [personal communication]. Overall, these data will be informative to develop thresholds for analysis of mtGenome MPS data.

### 3.4. Accuracy

Strobl et al. [24] described the analysis and accuracy of haplotype calls generated for 521 samples across 24 different populations. Their study showed the performance of the Precision ID mtDNA Whole Genome Panel across all major clades of the mtGenome phylogeny (except O and S), supporting that sequence motifs specific to phylogenetic backgrounds had little effect on primer binding or amplification efficiency. The accuracy study herein adds to that work with an additional 126 samples. These samples were sequenced in sequencing runs 13 through 16 (Table S1). Sequencing runs 13 and 14 were based on 300 pg of input DNA, and sequencing runs 15 and 16 were based on 1 ng of input DNA.

The 62 samples included in sequencing runs 13 and 14 and the 64 samples included in sequencing runs 15 and 16 each reached the 20X read depth threshold at 99% or greater of the nucleotide positions across the mtGenome. The specific ranges of sequence coverage at 20X or greater for these 126 samples are provided in Table S5. Six samples from sequencing runs 13 and 14 (A14, A17, A22, A41, A42, and A46) and seven samples from sequencing runs 15 and 16 (A70, A89, A95, A99, A102, A107, and A111) had regions of the mtGenome drop below the 20X read depth threshold. A SNP was identified in the primer binding region of each of these lower performing amplicons, which likely is the factor in the reduced amplification efficiency. Regions of the mtGenome that failed to reach the 20X read depth threshold (Table S3) were not included when evaluating concordance.

Haplotype calls for each of the samples in the accuracy study are provided in Table S5. Previously generated data were available for 124 of these 126 samples for an evaluation of concordance. Haplotypes from the samples included in sequencing runs 13 and 14 were compared to mtGenome data previously generated on the MiSeq [10]. A total of 3574 sequence variant calls were made across the 62 samples sequenced in runs 13 and 14, and 43 (1.2%) of these sequence variant calls were discordant with the MiSeq data (Table S6). Thirty-three (76.7%) of these discordant calls included deletions called at the end of long C stretches in hypervariable region I (HVI) and hypervariable region II (HVII) (i.e., 309del, 315del, and 16193del) in S5 data and not identified in the MiSeq data, indicating possible false deletions above the 40% deletion threshold used for analysis. Two (4.7%) of the discordant calls were insertions that fell below the threshold and, thus, were called in only the S5 data (*n* = 1) or only the MiSeq data (*n* = 1). Two variant calls were marked discordant due to a difference in the dominant haplotype for the inserted nucleotides. One point heteroplasmy was called in only the S5 data as reads with the alternate haplotype comprised less than 1% of the total read depth in the MiSeq data. One substitution (8794T) was called only in the MiSeq data. In this sample, the amplicon (mt_85) had a low read depth (34X), likely affected by the SNP located in the primer binding region at nucleotide position 8896. Finally, four potential sequence variants in the S5 data were labeled with an “N” due to a difficulty in differentiating between a true potential mtDNA variant or nuclear mitochondrial DNA (NUMT) reads, confirming results described by Sturk-Andreaggi et al. [16] where data from short amplicon and long-PCR workflows were compared.

Haplotypes from 62 of the samples included in sequencing runs 15 and 16 were compared to previously generated whole (*n* = 24) or partial (*n* = 38) mtGenome Sanger sequence data. A total of 2381 sequence variant calls were made across the 64 samples sequenced in runs 15 and 16. Seventy-five (3.1%) of these sequence variant calls were discordant with the Sanger data, and 73 (97.3%) of the discordant calls occurred in homopolymeric regions (Table S6). Thirty-four (45.3%) of the discordant calls included deletions called at the end of long C stretches in HVI and HVII (i.e., 309del and 16193del) in S5 data and were not identified in the Sanger data, again indicating potential false deletions above the 40% deletion threshold. Twenty (26.7%) of the discordant calls were insertions called in the Sanger data only, although reads with insertions were present but did not meet the 30% threshold in the S5 data. Five (6.7%) variant calls were marked discordant due to a difference in the dominant haplotype for the inserted nucleotides. Fifteen variant calls (20.0%) were point heteroplasmies called in only the S5 data, and one point heteroplasmy (1.3%) was called only in the Sanger data as the MPS reads with the alternate haplotype did not reach the 10% threshold.

A 309del variant initially was called in 58 of the 126 (46.0%) accuracy study samples using the thresholds set for analysis in this study. Each of the 309del calls were discordant with MiSeq or Sanger sequencing concordance data (Table S6). However, based on the concordance data and EMPOP data [1], which indicate that 309del is a rare variant, these 309del calls were considered false deletions and not included in the final haplotype calls in Table S5. Data from the accuracy study provide valuable information for laboratories to consider when setting thresholds for MPS analysis. Data show special attention is needed in homopolymeric regions and higher thresholds for deletions may need to be implemented. In addition, the results illustrate the current need for manual verification of haplotype calls by a trained analyst.

### 3.5. Sensitivity

To determine the reliability of results from a range of input DNA quantities, serially diluted DNAs from 12 distinct samples were sequenced (runs 17 through 20; Table S1). While the mtGenome has a higher copy number per cell [56,57] relative to the two copies of nuclear DNA per cell, mtGenome copy number can vary among sample type, tissue type, and individual being sampled, making nuclear DNA quantifications at times a poor surrogate for assessment of mtGenome copy number. Despite this limitation for template quantification, a number of laboratories still use a nuclear DNA quantification method to determine the amount of input DNA. Therefore, this study included serial dilutions based on nuclear DNA quantities (runs 17 and 18; Table S1) and mtGenome copy number (runs 19 and 20; Table S1).

Dilutions were prepared by serially dividing the concentration of DNA in half six times for a total of seven DNA quantities. The dilutions represented input amounts of nuclear DNA ranging from 150 pg to 2.3 pg, and mtDNA quantities ranging from 1500 mtGenome copies to 23.5 mtGenome copies. As these library preparations were automated on the Ion Chef, these DNA input amounts represent total DNA input amounts for both primer pools (i.e., half the input DNA went into each PCR).

Read depth at each nucleotide position was averaged across the mtGenome for these serially diluted samples to determine the difference in the number of reads generated for the differing inputs of DNA (Table S7). As expected, with each consecutive reduction in DNA input amount, the average read depth decreased as well. The potential variability in mtDNA copy number between individuals is illustrated in Figure S3A,B, where the average read depth for each point of the dilution series in all 12 samples is compared. The standard deviation of the average read depth for each DNA input amount was calculated across the six dilution series and found to be smaller at each data point for the mtDNA quantifications. The samples with mtDNA quantifications were able to produce more consistent results across the dilution series compared to samples with input based on nuclear DNA quantifications. Additionally, the percent of the mtGenome that reached the 20X read depth threshold set for analysis in this study was calculated for each sample at each DNA input amount. Each sample had more than 90% of the mtGenome reach the 20X read depth threshold (Table S7), with those regions below 20X primarily attributed to the decreasing DNA input amounts. Sample SS1 at 2.3 pg of input DNA and SS4 at 9.4 pg of input DNA had regions of the mtGenome that dipped below the 20X read depth threshold (Table S7). For sample SS11, data from the five DNA input amounts also showed a small region of the mtGenome that did not reach the 20X read depth threshold. This region was consistent across all five samples (mt_3) and likely a result of the two SNPs in a primer-binding site, at nucleotide position 225 and 227, for this amplicon.

DNAs from the 12 distinct samples in the sensitivity study also were used as part of the accuracy study, where haplotypes were compared to data generated on an orthogonal platform to assess concordance. The haplotypes from the accuracy study were used to assess stochastic variation at the different quantities of input DNA in the sensitivity study. PCR amplification of low levels of DNA can lead to stochastic effects, which refer to an un-even sampling of the DNA molecules present in the PCR [58,59,60]. While the ability to produce full mtGenomes (i.e., read depth of at least 20X) remained relatively consistent across the DNA input amounts, the sensitivity, or ability, to distinguish true mtDNA variation from other sources of reads, such as exogenous DNA or NUMT reads, decreased as DNA input amounts decreased. Therefore, additional sequence variants not part of the true mtDNA haplotype were called at lower DNA input amounts (labeled as stochastic variation in Table S7). This stochastic variation was found at nuclear DNA input amounts of 18.8 pg or less and at input amounts of 93.7 mtGenome copies or less.

**Figure 6:**
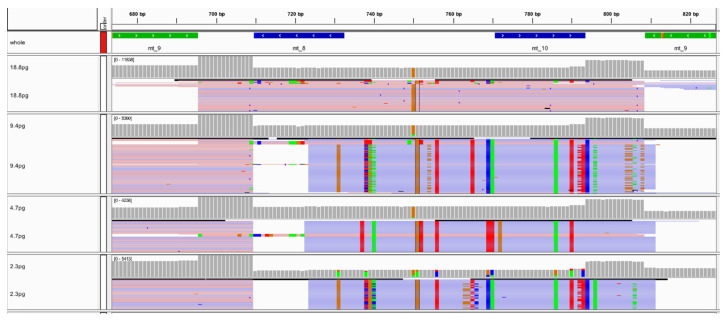
Screenshot from IGV illustrating the increasing number of nuclear mitochondrial DNA (NUMT) reads aligned to amplicon mt_9 as the amount of input DNA decreased.

Two additional trends in the dilution series’ data can likely explain some of the stochastic variation that was identified at lower template input amounts. A no template control (NTC) was prepared with each dilution series that was generated for this study. The relative read depth for the NTC was calculated as described in the contamination study in reference to the read depth generated for each DNA input amount in each dilution series. This value was averaged across the entire mtGenome (Table S7). Based on these values and the thresholds used for data analysis in this study, the potential for amplification of exogenous DNA having an effect on variant calling increases as the amount of input DNA decreases, particularly at 23.5 mtGenome copies or nuclear DNA amounts of 4.7 pg or less. Secondly, the number of NUMT associated variants (NAVs) that reached the 10% point heteroplasmy threshold increased as the amount of input DNA decreased (Figure 6). The higher copy number of mtDNA relative to nuclear DNA typically leads to a higher read depth of true mtDNA variation relative to any possible NUMT associated variation and helps identify NUMT reads. However, this trend does not hold in situations of decreased amplification efficiency [55] or lower DNA input amounts, especially with amplicons that have been inserted into the nuclear genome multiple times. Additionally, while reads with high homology to a nuclear chromosome insert were readily identifiable and labeled as “false” by Converge, NUMT reads that differ by only one SNP variant can be more difficult to distinguish from true mtDNA variation [55]. Each of these trends, reads from exogenous DNA contamination and NUMTs increasing relatively with decreasing input DNA, combined with the possibility of allele drop-in, suggests that higher point heteroplasmy thresholds for lower DNA input amounts may need to be implemented.

### 3.6. Mock Casework Samples

The mock casework study focused on sequencing sample types regularly encountered in forensic investigations. Sequencing runs 21 through 28 (Table S1) included DNA extracted from hairs, bones, buccal swabs, blood, semen, saliva, and blood stains. Each of the samples included in this study went through three different library preparation methods (automated on the Ion Chef, manual, and manual with an additional library amplification step; Table S1) [36]. Results from each of these workflows were compared to previously generated Sanger sequence data for the control region of the mtGenome.

**Figure 7:**
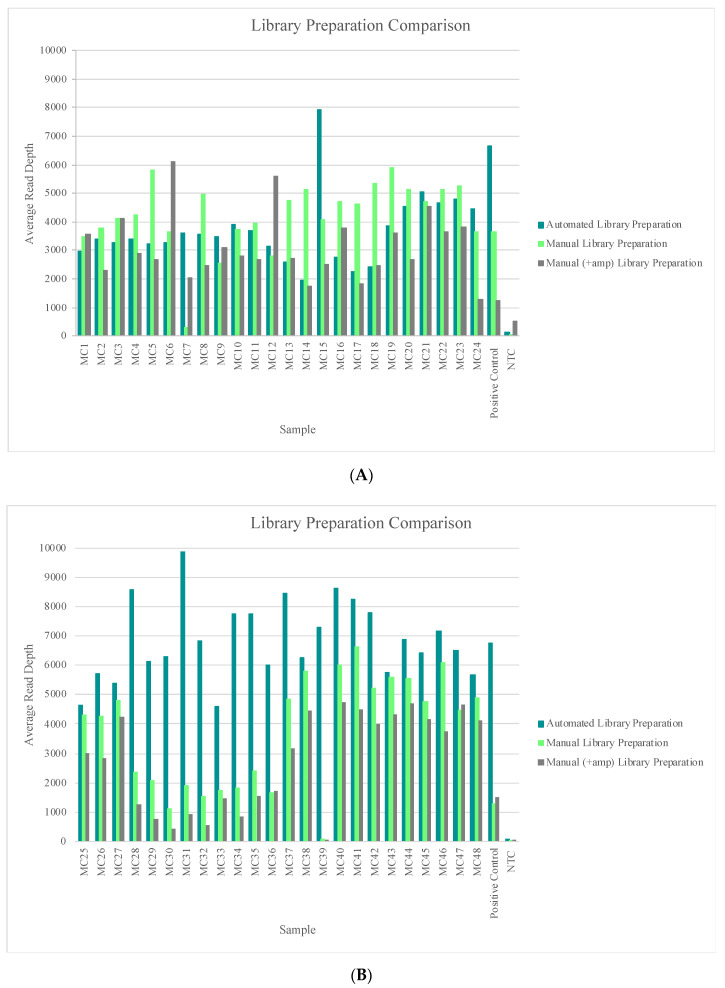
(**A**) The average read depth across the mtGenome for each sample included in sequencing runs 21 through 24 (completed by laboratory 1) is illustrated. Results were sorted by the library preparation method for an assessment of read depth. (**B**) The average read depth across the mtGenome for each sample included in sequencing runs 25 through 28 (completed by laboratory 2) is illustrated. Results were sorted by the library preparation method for an assessment of read depth.

Samples included in this study again illustrate performance on a variety of nuclear DNA input amounts (sequencing runs 21 through 24, Table S1) and on a variety of mtDNA input amounts (sequencing runs 25 through 28; Table S1). The hair, bone, and buccal swabs included in sequencing runs 21 and 22 were generated from DNA input amounts ranging from “undetermined” to 300 pg of nuclear DNA, and all of these samples reached the 20X read depth threshold at 100% of the nucleotide positions across the mtGenome. The blood, semen, saliva, and blood stain samples included in sequencing runs 25 and 26 were generated from DNA input amounts of 100 mtGenome copies or 1000 mtGenome copies, and all of these samples reached the 20X read depth threshold at 100% of the nucleotide positions across the mtGenome. Figure 7 shows a comparison of each samples’ performance across the three library preparation methods used in this study. The read depth at each nucleotide position was averaged across the mtGenome for each sample in sequencing runs 21 through 28. The trends seen in Figure 7A,B represent samples that were included in different sequencing runs completed by different laboratories (Table S1), which can account for the variability seen between the two figures. However, in both Figure 7A,B, the automated library preparation methodology lacked the punctuated dips in sample performance (average read depth) that were seen with the manual methods (e.g., MC7 and MC39). These lower-performing samples likely were the result of user-introduced variability during the workflow and highlight one of the benefits of automation.

The haplotype calls for the 48 mock casework samples included in this study are provided in Table S8. Concordance was evaluated across the entire mtGenome for the data generated from all three library preparation methods. If a sequence variant reached the thresholds used for analysis in at least one of the haplotypes generated from the three library preparation methods, the variant was included in the final haplotype provided for the mock casework samples in Table S8. A total of 1755 sequence variant calls were made across the 48 mock casework samples, and 37 of these sequence variants (2.1%) did not receive the same call for all three library preparation methods. Sixteen of these 37 variant calls (43.2%) were from the haplotypes generated from sample MC39. The lower performance of MC39 for the manual library preparation methods is shown in Figure 7B and likely is a factor in the variability of the haplotype calls produced from data generated by these three library preparation methods. The average read depth across the mtGenome for MC39 was 7295X (±3783X), 74X (±57X), and 58X (±44X) for the automated, manual, and manual plus amplification library preparation methods, respectively. As seen in previous studies, lower performing samples (whether from the library preparation process, amount of input DNA, etc.) can result in a decrease in read depth and a subsequent decrease in sensitivity or ability to distinguish true mtDNA variation from other sources of reads (i.e., exogenous DNA or NUMTs). The remaining 21 sequence variants were present in the data from each library preparation method but did not reach the thresholds for analysis in each instance. These 21 sequence variants included: 309.1C in MC5, MC7, MC25, MC26, MC33, MC36, MC38, MC47, and MC48; 309del in MC11, MC12, MC32, and MC44; 309.2C in MC6 and MC14; 315del in MC42; 16192del in MC41; 16182M in MC 41; 16183M in MC29 and MC34 and 12275M in MC5. Only one of these (12275M) was not located in long homopolymeric regions of HVI and HVII. Additionally, the 309.1C sequence variant was not called by Converge for each of MC28, MC39, and MC38’s haplotypes, and the 13617Y sequence variant was not called by Converge for each of MC31’s haplotypes despite sequence variant frequencies over the related insertion or heteroplasmy threshold due to the strand bias threshold set in Converge. Each of these was identified and called during manual review of the haplotypes.

Next, the “consensus” haplotypes for the 48 mock casework samples were compared to previously generated Sanger sequencing data for the control region. Thirty-seven (2.1%) instances of discordance were identified and included in Table S9. Two of these discordances were sequence variants identified in only the MPS haplotype (309.1C in MC8 and 16186Y in MC9). However, these sequence variants were found to be present in data from both technologies when the Sanger data were re-reviewed, bringing the total number of discordances down to 35. Five (14.3%) more of these discordances were sequence variants identified in only the Sanger haplotypes. Each of these five sequence variants were present in the MPS data, but they did not reach the thresholds used for analysis. Twenty-five (71.4%) of these discordant calls included deletions called at the end of long C stretches in HVI and HVII (i.e., 309del, 315del, 16192del, and 16193del) in MPS data and not identified in Sanger data, highlighting possible identification of false deletions above the 40% deletion threshold used for analysis. The remaining discordances were point heteroplasmies called only in the MPS data. This potentially could reflect an increase in sensitivity and resolution with the MPS technology’s ability to detect low-level variation. However, the low performance of at least one sample (MC39) must also be taken into account.

Six (17.1%) of the discordant sequence variant calls were found in data for MC39. The low performance, and thus, low sensitivity of the data generated by two library preparation methods for MC39 are potential factors in these discordant calls. At lower read depths, stochastic variation or amplification of NUMTs or exogenous DNA can be difficult to discern and, thus, cannot be ruled out for MC39. During manual review of the data, all identifiable NUMT associated variants (NAVs) were removed. In instances of low sample performance, poor amplification efficiency, or amplicons with high homology to the associated NUMT insert, NAVs can be difficult to identify [55]. An 8943N was included in the haplotype for MC46. When viewing data for this sample, two SNPs were identified in the primer-binding region of amplicon mt_86 at nucleotide positions 8860 and 8869, which likely contributed to the lower read depth for this amplicon relative to samples without SNPs in the primer-binding region. Reads with a T nucleotide at position 8943 were present at 69% of the total read depth or greater for each MPS haplotype. However, 8943T was not listed in V1a1 or its subgroups in EMPOP [1], and reads with a T nucleotide at position 8943 exhibited primary homology to chromosome one (University of California Santa Cruz (UCSC) BLAT tool) [61]. This information combined with the poor amplification efficiency suggested 8943Y in MC46 was a possible NAV [55]. Therefore, 8943N was included in the MC46 haplotype.

A 309del variant initially was called in 19 of the 48 (39.6%) mock casework samples using the thresholds set for analysis in this study. Each of the 309del calls were found to be discordant with Sanger sequencing concordance data (Table S9). However, based on concordance data and EMPOP data [1], which indicate that 309del is a rare variant, these 309del calls were considered false deletions and not included in the final haplotype calls in Table S8. Data from the mock casework study provide valuable information for laboratories to consider when setting thresholds for MPS analysis, suggesting higher thresholds for low-performing samples and deletions may need to be implemented. Mock casework study data also showcase examples where manual verification of haplotype calls by a trained analyst is still necessary.

### 3.7. Mixtures

Churchill et al. [40] previously described the sequencing and analysis of two-person and three-person mixtures with contributors of similar and differing phylogenetic backgrounds using the Precision ID mtDNA Whole Genome Panel, Ion Chef, and Ion S5/Ion PGM workflow. Therefore, the artificial mixtures prepared for this study were designed to go beyond the previous study by diluting the amount of input DNA of components of the mixture almost to extinction. Sequencing runs 29 through 32 (Table S1) included artificially mixed samples of known haplotypes at a nuclear DNA ratio of 20:1, 10:1, 5:1, 2:1, 1:2, 1:5, and 1:10. One contributor was kept at a constant DNA input amount of 2 pg while the second contributor’s amount of input DNA varied from 40 pg to 0.2 pg in order to generate the targeted mixture ratios. DNA from different contributors was used for each of the four mixture series included in sequencing runs 29 and 30 (Table S1), while the same contributors were used for each of the four mixture series included in sequencing runs 31 and 32 (Table S1). Due to the higher variability and differing thresholds used for analysis, indels were not included in attempts to deconvolute the mixed haplotypes.

**Figure 8:**
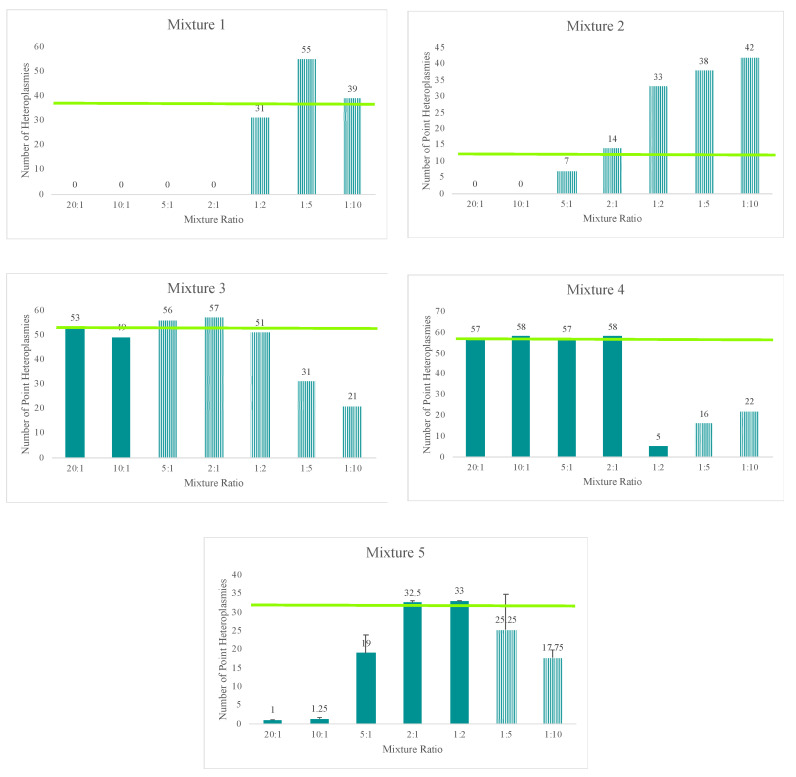
The number of mixed variant point sites (MVs) for each mixture ratio in the five mixture series (averages are provided for the fifth mixture series as this series was run four times in sequencing runs 31 and 32) included in this study. Given the known haplotypes of each contributor, the expected number of MVs is indicated on the graphs by the horizontal bar. Furthermore, given the known haplotypes of each contributor, mixtures with stochastic variation present in the mixed haplotype are indicated by the striped bar.

The total amount of input DNA for the mixtures prepared in this study ranged from 42 pg (20:1 mixture) to 2.2 pg (1:10 mixture). Despite the low DNA input amounts, each of the mixtures reached the 20X read depth threshold at 97% or greater of the nucleotide positions across the mtGenome. A slight decrease in sensitivity, or ability to sequence the entire mtGenome at a read depth of 20X or greater, was seen at total DNA input amounts of 3 pg or less. The number of nucleotide positions with two or more allele states (mixed variant point site or MV) present in a haplotype offers an informative metric for identifying the presence of a mixed haplotype. Thus, the number of MVs for each mixture in the five mixture series (averages are provided for the fifth mixture series as this series was run four times in sequencing runs 31 and 32) included in this study are provided in Figure 8. Given the known haplotypes of each contributor, the expected number of MVs is indicated on the graphs by the horizontal bar. The presence of three of more (to allow for personal point heteroplasmies) MVs was used to indicate the presence of a mixture. Although nuclear DNA quantifications were used to generate the targeted mixture ratios, trends were consistent; the more similar the mtDNA input of each contributor, the more likely to see sequence variants from both contributors in the mixed haplotype. Since mtDNA content can vary between individuals, the actual ratio of contributor DNAs varied from those targeted and is discussed in more detail below. Using the average sequence variant frequencies of the major and minor contributor as a surrogate for calculating the ratio of mtDNA from each contributor, the number of MVs indicated the presence of a mixture at ratios of 9:1 or less. The mixtures with more disparate mtDNA quantities of each contributor (e.g., 20:1) had three MVs or less, suggesting only the major contributor’s haplotype would be identified above the 10% heteroplasmy threshold (e.g., mixture series one in Figure 8).

The mixtures’ decreasing amounts of input DNA, ranging from 42 pg to 2.2 pg, resulted in two additional trends when examining the total number of MVs for each mixture (Figure 8). A loss of sensitivity, or ability to detect the full minor contributor’s haplotype above the 10% heteroplasmy threshold, was seen with decreasing amounts of input DNA (e.g., mixture series three in Figure 8). Stochastic effects also were encountered when amplifying lower DNA input amounts, resulting in more variable sequence variant frequencies. These stochastic effects combined with the decreasing sensitivity to distinguish between true mtDNA variation or amplification of NUMTs or potential exogenous DNA at lower DNA input amounts lead to the inclusion of more MVs beyond that of the known profiles of each contributor at 12 pg of total input DNA or less, consistent with the results seen in the sensitivity study (Figure 8).

**Figure 9:**
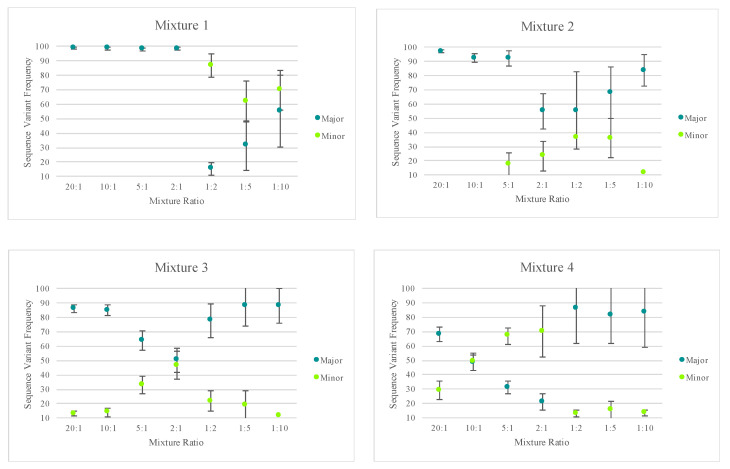

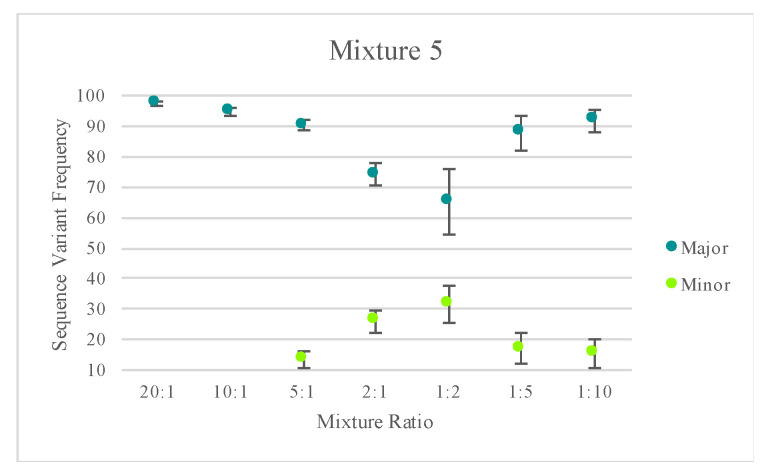
The average sequence variant frequency (and standard deviations) for the major and minor contributor included in each mixed haplotype. Only one of the replicates for mixture series 5 was included in order to allow the standard deviations to reflect variability in the sequence variant frequencies of one mixture instead of reflecting the variability across several mixtures. The Y-axis starts at 10 to reflect the 10% point heteroplasmy threshold used for analysis.

A similar analytical strategy to that described in Churchill et al. [40] was used to illustrate the potential for mixture deconvolution. The quantitative nature of MPS data offers opportunities to quantitatively separate the haplotypes of each contributor to a mixture using the sequence variant frequencies (Figure S4). However, factors such as the quantity of each contributor, NUMTs, stochastic variation, amplification efficiency, etc. can impact the efficiency of deconvolution (example illustrated in Figure S5). The average sequence variant frequency (and standard deviations) for the major and minor contributor included in each mixed haplotype were calculated and are displayed in Figure 9. Only one of the replicates for mixture series five was included for calculating the standard deviations to reflect variability in the sequence variant frequencies of one mixture instead of reflecting the variability across several mixtures. Figure 9 highlights the difference in the targeted nuclear DNA ratios of each mixture versus the actual ratio of mtDNA content in each mixture. These values varied across each mixture series as the mtDNA copy number can differ among individuals. For two of the mixture series, one contributor’s mtDNA content was notably higher than the second contributor’s, causing the minor contributor to appear similar to the major contributor in terms of sequence variant frequency/read depth. Nonetheless, quantitative data could be used to successfully deconvolve mixtures with notable differences between the contributors.

With a heteroplasmy threshold of 10%, MVs representing the haplotypes of the two known contributors were observed in the majority of the mixed samples and were quantitatively distinguishable. Using the sequence variant frequencies of the major and minor contributor as a surrogate for calculating the ratio of mtDNA from each contributor, mixture ratios greater (or more disparate) than 1:9 exhibited only the major contributor’s profile above the 10% heteroplasmy threshold. True mtDNA mixture ratios of approximately 1:1 (i.e., 2:1 mixture in mixture series three and 10:1 mixture in mixture series four) were too similar to be quantitatively separated. For these mixtures, additional phylogenetic or phasing information could assist in the deconvolution of some mixtures. The number of MVs detected above the thresholds varied among the remaining mixture ratios. Mixed haplotypes with mtDNA contributions at ratios ranging from 1:2 to 1:9 were able to be quantitatively separated, leading to identification of the major contributor’s haplotype and the minor contributor’s partial or full haplotype. However, stochastic effects and a decreasing sensitivity for true mtDNA variation over amplification of NUMTs or exogenous DNA at total DNA input amounts of 12 pg or less made identification of the minor contributor’s full haplotype difficult. The known profiles of each contributor were used to assess presence or absence of each expected MV and the frequency of the contributor’s haplotype (Figure 9). Stochastic variation likely added to the increasing standard deviations seen in the mixtures with lower amounts of input DNA (Figure 9).

There was no set ratio of reads from the major and minor contributors used in this study to quantitatively deconvolve mixtures. Such thresholds should be determined with internal validations studies. While not a factor in previous studies [40], the stochastic variation present in mixtures of lower DNA input amounts (12 pg total or less in this study) made parsing more difficult. The potential presence of stochastic variation will have to be considered as laboratories develop thresholds for analysis. While three or more MVs were used in this study to indicate the presence of a mixture, further work is needed to determine the number of expected heteroplasmic positions in whole mtGenome data from single-source samples, which is likely to vary by sample type, age, and threshold [41,62,63]. Historically, mtDNA mixtures have not been interpreted in forensic laboratories, hindered, at least in part, by Sanger sequencing’s lack of quantitative information to parse apart contributors. Results from this study indicate that this MPS approach aids in the interpretation of mixed mtDNA sequences compared to analysis with current CE technologies. With continued bioinformatic developments, including probabilistic genotyping approaches, mtDNA mixture analysis will become more robust and likely more routine for forensic laboratories.

### 3.8. Species Specificity

**Figure 10:**
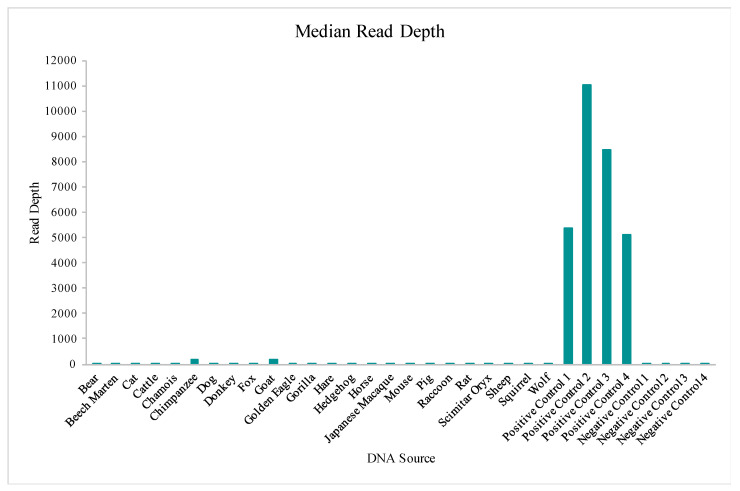
The median read depths aligned to the revised Cambridge Reference Sequence (rCRS) for each of the 24 vertebrate DNAs, four positive controls, and four negative controls included in the species specificity test are displayed.

**Figure 11:**
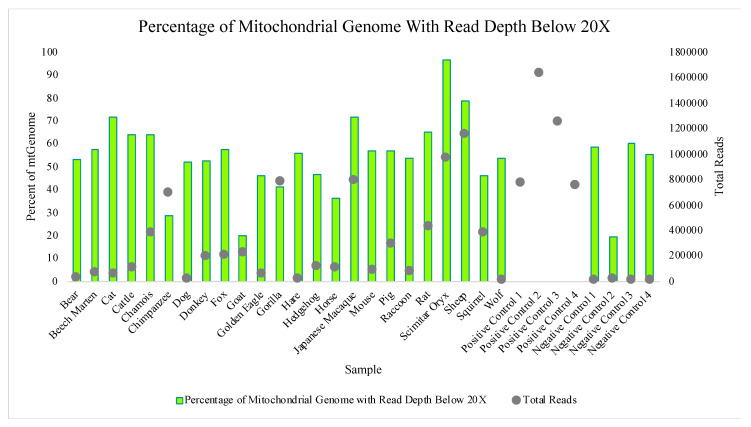
The percentages of nucleotides aligned to the rCRS with a read depth below 20X for each of the 24 vertebrate DNAs, four positive controls, and four negative controls included in the species specificity test are displayed. The scatter-plot points show the total number of reads generated for each sample.

The Precision ID mtDNA Whole Genome Panel’s amplification cross reactivity with non-human species was evaluated using DNA extracts from 24 different vertebrates, including bear, beech marten, cat, cattle, chamois, chimpanzee, dog, donkey, fox, goat, golden eagle, gorilla, hare, hedgehog, horse, Japanese macaque, mouse, pig, raccoon, rat, scimitar oryx, sheep, squirrel, and wolf DNA. Details on this sequencing run can be found in Table S1. A total of four positive control (human DNA) and four negative control samples also were included in this run for comparative evaluations of performance. While aligned reads were observed for each sample included in this study, median read depths for each of the 24 vertebrate DNAs were more consistent with those of the negative controls versus the positive controls included in the run (Figure 10), supporting that these reads may be due more to background DNA than to alignment with the species genome. This similarity to the negative controls is illustrated again when evaluating the number of nucleotide positions across the mtGenome, where the read depth failed to reach the 20X threshold (Figure 11). The percentage of rCRS with nucleotide positions below 20X ranged from 20% (goat DNA) to 97% (scimitar oryx) for the 24 vertebrate DNAs, again suggesting the low read depth across much of the mtGenome could be due to human molecules contaminating the samples during collection [64]. Chimpanzee and goat DNA exhibited the most nucleotides where the 20X read depth threshold was reached.

Based on median read depth values and the portion of the mtGenome with a read depth above 20X, the chimpanzee and goat results were analyzed further. The National Center for Biotechnology Information (NCBI) BLAST results [65] showed a 91.33% and 76.6% identity when comparing the mitochondrial genomes of the chimpanzee and goat, respectively, to the human mtGenome. In fact, homologous sequences in both the goat and human mtGenomes contain the primer regions for amplicons mt_14 and mt_15, which explain the two strong read depth peaks (Figure S6). When analyzing additional vertebrate DNAs, some level of homology was expected due to the short distances between the primer pairs used to amplify the mitochondrial genome in this study. However, similar analytical strategies, such as those used to address NUMTs [55,66], can aid in addressing amplicons that exhibit potential for amplifying additional species, if there would be any relevance. These strategies include inconsistencies in relative read depth results, primary homology to different genomes, and inconsistencies in the phylogeny results—as used below in evaluating the chimpanzee and goat DNA samples.

**Figure 12:**
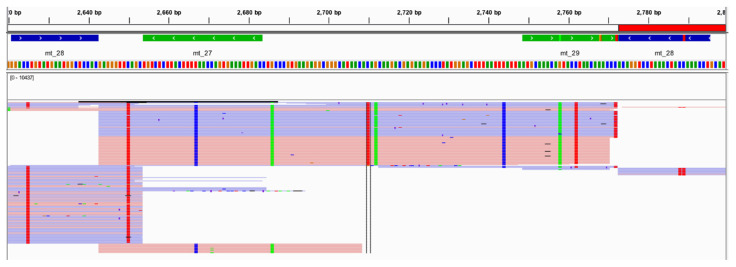
An example of the alignment and variant calling results, shown in IGV, for the chimpanzee at one amplicon (mt_28) illustrating the presence of several sequence variants in one amplicon.

**Figure 13:**
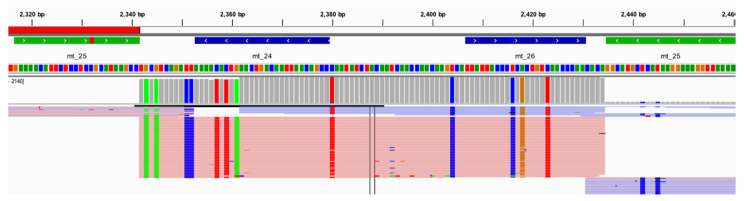
An example of the alignment and variant calling results, shown in IGV, for the chimpanzee at one amplicon (mt_25) illustrating extreme strand bias (>89).

Multiple studies have displayed similar relative read depth patterns for the amplicons generated by the Precision ID mtDNA Whole Genome Panel. However, when looking at the read depths of the 24 vertebrates evaluated in this study, differing relative read depth patterns appear (Figure S6). The read depths for the reads amplified from chimpanzee DNA and aligned to rCRS ranged from 0X to 90,724X. A total of 1063 SNPs and indels were identified by Converge when aligning the raw data generated from chimpanzee DNA to rCRS. This outcome represents approximately ten times more nucleotide differences from the rCRS reference genome than the number of pairwise differences identified within and between the major US populations in previous studies [10], raising a red flag for the evidence sample genome aligned to a human reference. In fact, UCSC Genome Brower BLAT results [61] for one randomly selected amplicon (Figure 12) illustrated primary homology to chimpanzee. Additionally, 131 (12.6%) of these sequence variants identified by Converge were heterozygous, and 228 (21.4%) of these sequence variants exhibited extreme strand bias (>0.89; Figure 13), raising additional flags for the sample not being human in origin. A total of 250 (23.5%) sequence variants were labeled by Converge as “Unknown in EMPOP”, suggesting a different phylogeny from the well-characterized human phylogeny present in EMPOP [1]. Similar results were observed for the goat DNA sample. Read depths for the reads amplified from goat DNA and aligned to rCRS ranged from 0X to 53,069X across the alignment with rCRS. A total of 110 SNPs and indels were identified by Converge when aligning the raw data generated from goat DNA to rCRS, which is still larger than the number of pairwise differences identified within and between major US populations [10]. Of these sequence variants, 55 (50%) variants were labeled heterozygous by Converge, and 21 (19%) of these variants exhibited extreme strand bias. A total of 77 (77%) were labeled by Converge as “Unknown in EMPOP” or “Unexpected” in the closest haplogroup call for the evaluated data.

Despite the number of nucleotide positions in some samples that reached the 20X read depth threshold used for analysis, the data presented opportunities to identify the presence of non-human DNA. Overall, the Precision ID mtDNA Whole Genome panel exhibited the expected degree of specificity to human mtDNA and bioinformatic analyses can reduce potential alignments from other species being interpreted as being from a human source.

## 4. Conclusions

The study herein describes the developmental validation study of the Precision ID mtDNA Whole Genome Panel, Ion Chef, Ion S5, and Converge workflow. This study builds on the work described in Strobl et al. [24] and Churchill et al. [40] and assesses the workflow’s reproducibility, repeatability, accuracy, sensitivity, specificity to human DNA, and ability to analyze challenged samples in accordance with the SWGDAM’s Validation Guidelines for Forensic DNA Analysis Methods [46]. Overall, the data produced in this study indicate that robust, reliable, and reproducible results can be achieved with the Precision ID mtDNA Whole Genome Panel, Ion Chef, Ion S5, and Converge workflow on samples of varying qualities and quantities. As such, the data indicate this workflow is a reliable method for generating mtDNA data for forensic applications. Currently, mtDNA control region data from the Precision ID mtDNA Whole Genome Panel have been approved for upload to the US National DNA Index System (NDIS) CODIS database (https://www.fbi.gov/services/laboratory/biometric-analysis/codis). The data herein should support expanding the mtDNA data uploaded to NDIS to the entire mtGenome.

This MPS workflow now makes it feasible to sequence and analyze the entire mtGenome for the broad spectrum of sample types encountered in forensic investigations. The increase in sensitivity over currently used CE-based technologies and the quantitative nature of the data provided by this MPS workflow allow for detection and characterization of lower-level heteroplasmies and avenues for mixture interpretation. However, data from this study also indicate that manual verification of haplotype calls by a trained analyst is still necessary. A trained analyst, familiar with the properties of the mtGenome, will help enable identification of potential NUMT reads, alignment errors, drops in read depth from primer-binding site SNPs, nomenclature errors, mixed samples, etc., to obtain highly accurate haplotype calls.

For forensic laboratories considering implementation of this workflow, quality assurance parameters and interpretation guidelines will be derived from their own internal validation studies. Data from this study will help drive the continued efforts and discussions needed to help generate guidelines for analytical thresholds. Future publications will address the development of these thresholds.

## Supplementary Materials

The following are available online at https://www.mdpi.com/2073-4425/11/11/1345/s1, Figure S1: Screen shot from IGV of the alignment difficulties in the mt_2 and mt_3 amplicon overlap with a 249del present, Figure S2: Screen shot from IGV of the reads from a negative control, Figure S3: Average read depth across the mtGenome for the dilution series samples, Figure S4: The sequence variant frequencies for one of the 2:1 mixtures included in sequencing run 31 were plotted against the nucleotide position of that sequence variant to illustrate the quantitative contribution of each contributor to the total read depth of the mixed haplotype, Figure S5: Screen shot from IGV illustrating the stochastic effects (i.e., variable sequence variant frequencies and drop-out) that can take place when amplifying lower amounts of input DNA, increasing the difficulty of deconvolution from these mixtures in mixture series 1 (sequencing run 29), Figure S6: Read depth for each nucleotide position across the rCRS reference genome for each of the 24 vertebrate DNAs, four positive controls, and four negative controls included in the species specificity run. Table S1: Summary of sequencing runs performed, Table S2: Summary of sequencing runs’ performance metrics, Table S3: Parameters used to configure HIDGenotyper plugin, Table S4: Consensus haplotypes generated for the samples and control DNAs sequenced in the reproducibility and repeatability study, Table S5: Haplotypes generated for the samples sequenced in the accuracy study, Table S6: Discordant sequence variant calls from the accuracy study, Table S7: Results of the sensitivity study showing the average read depth, percent of the mtGenome that reached the 20X read depth threshold, relative read depth of no template control (NTC), and whether stochastic variation was identified at each amount of input DNA, Table S8: Consensus haplotypes generated for the samples sequenced in the mock casework study, Table S9: Differences between the Sanger and MPS haplotypes.
