# Supplementary material for: Developmental Validation of a MPS Workflow with a PCR-Based Short Amplicon Whole Mitochondrial Genome Panel
Source: Genes (Basel). 2020 Nov 13;11(11):1345. doi: 10.3390/genes11111345 (PMC7709034; doi:10.3390/genes11111345)
Supplement: Supplementary file 1 [file genes-11-01345-s001.zip › genes-958458 supplementary/genes-958458-supplementary.pdf]

Supplementary Material

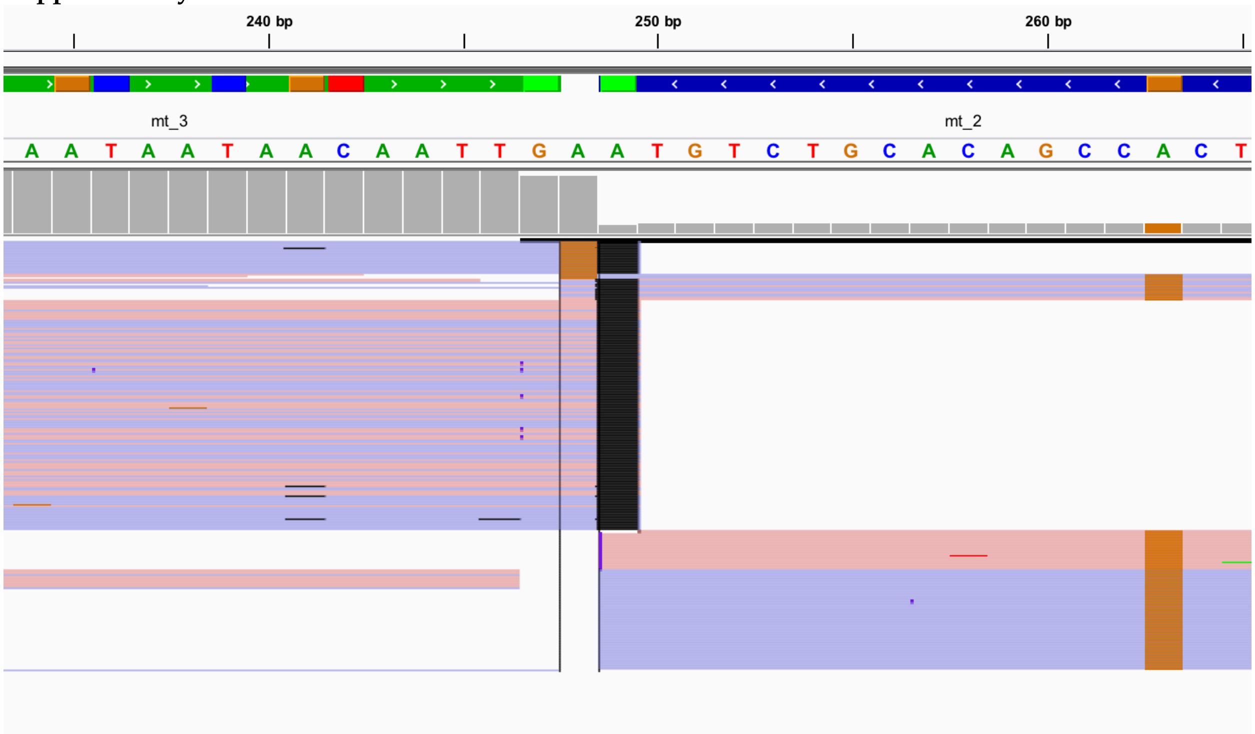

Figure S1. Screen shot from IGV of the alignment difficulties in the mt_2 and mt_3 amplicon overlap with a 249del present.

(A)

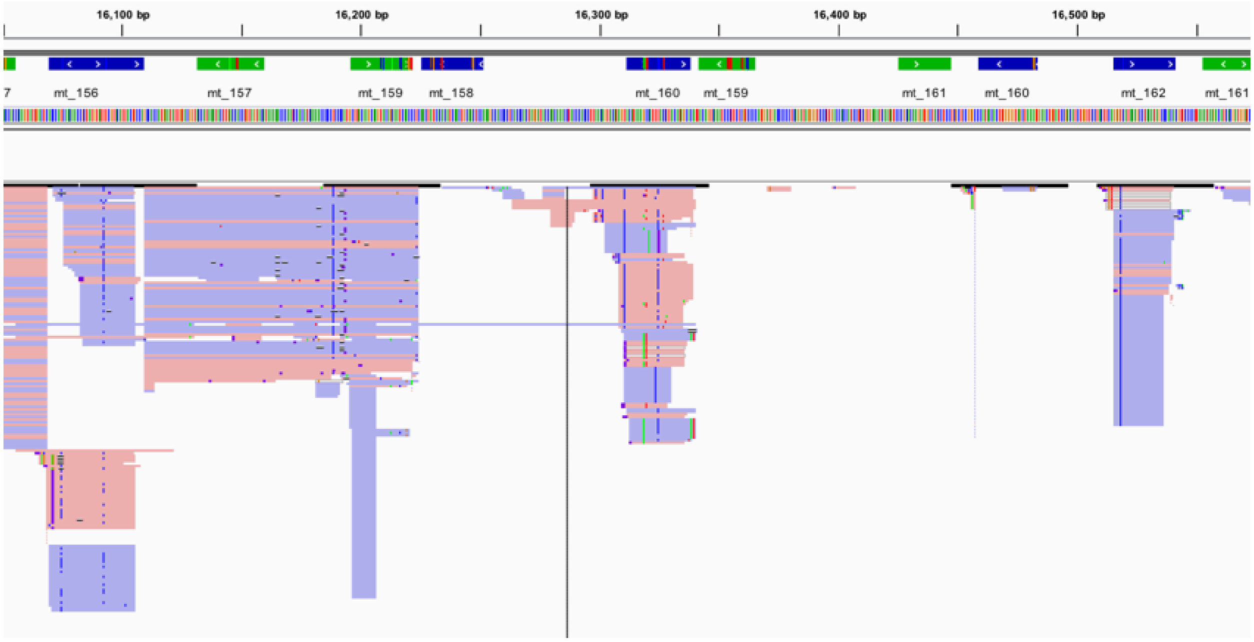

(B)

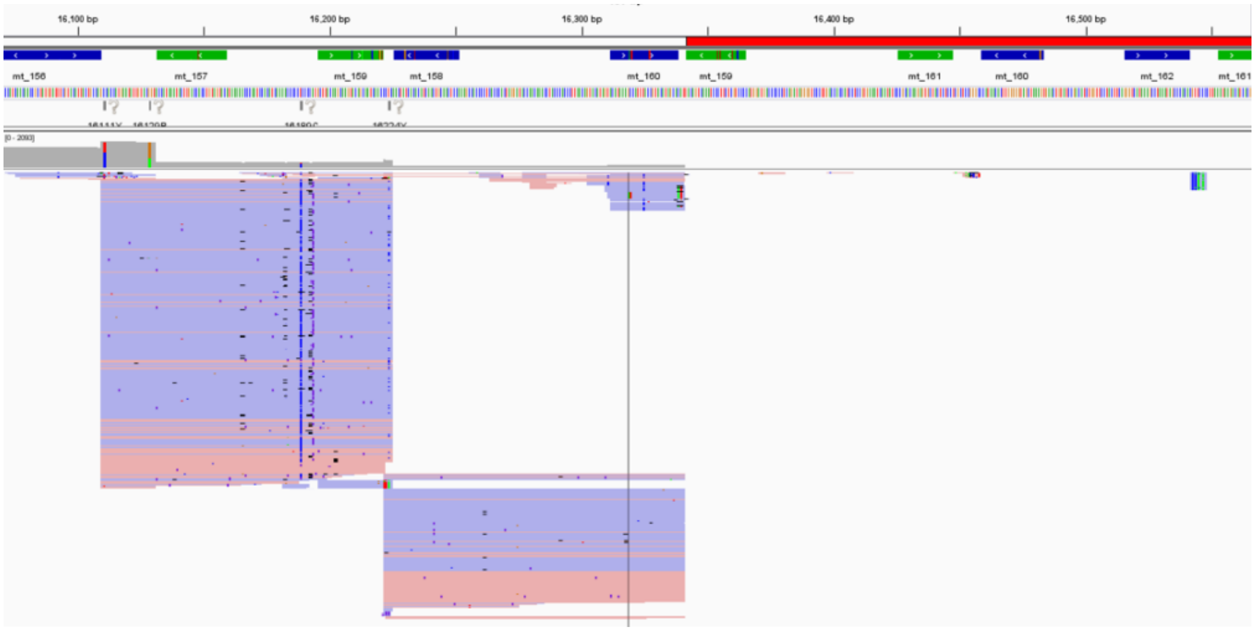
16,100 bp
16,200 bp
16,300 bp
16,400 bp
16,500 bp
mt_156
mt_157
mt_159
mt_158
mt_160
mt_159
mt_161
mt_160
mt_162
mt_161

(C)

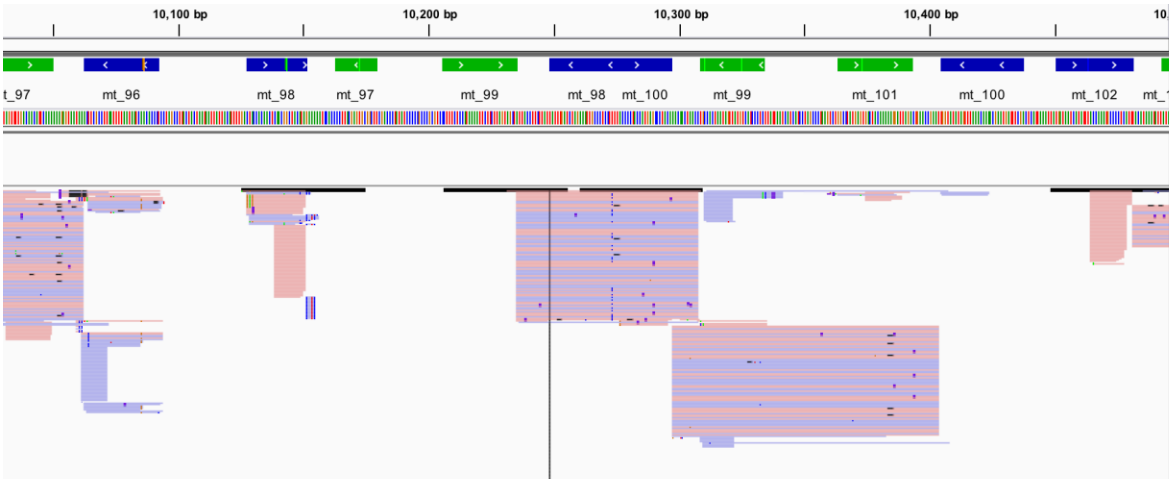
10,100 bp
10,200 bp
10,300 bp
10,400 bp
t_97
mt_96
mt_98
mt_97
mt_99
mt_98
mt_100
mt_99
mt_101
mt_100
mt_102

(D)

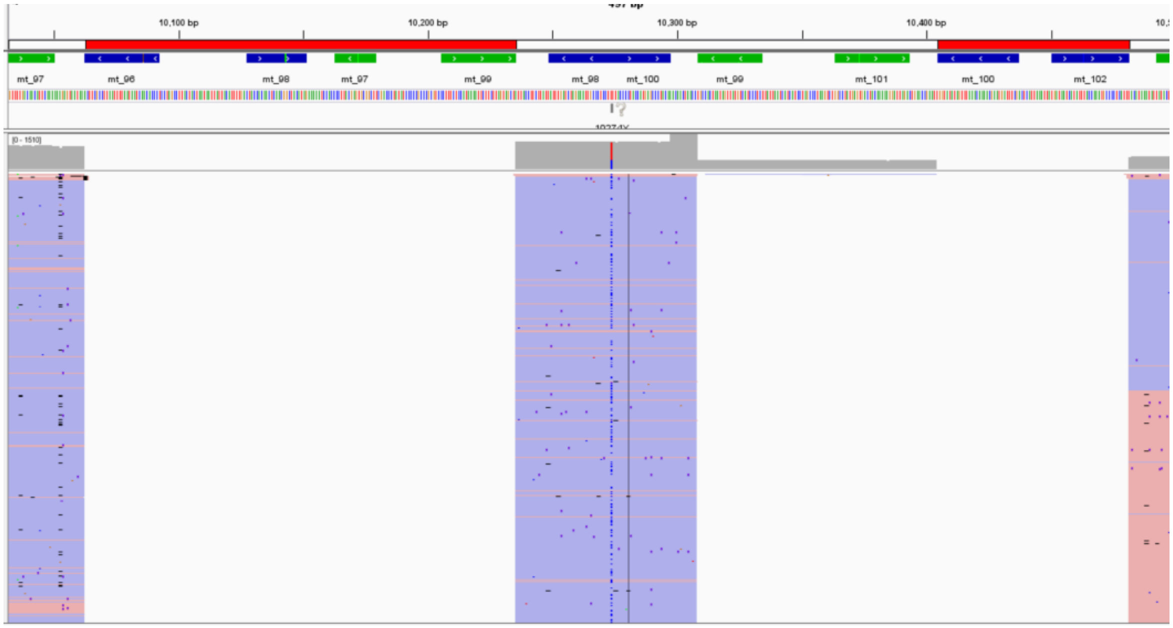

Figure S2. Screen shot from IGV of the reads from a negative control. A and B show the same region of the mtGenome prior to **(A)** and after **(B)** the latest Converge update. C and D show the same region of the mtGenome prior to **(C)** and after **(D)** the latest Converge update. The number of short reads is visibly less in B and D.

(A)

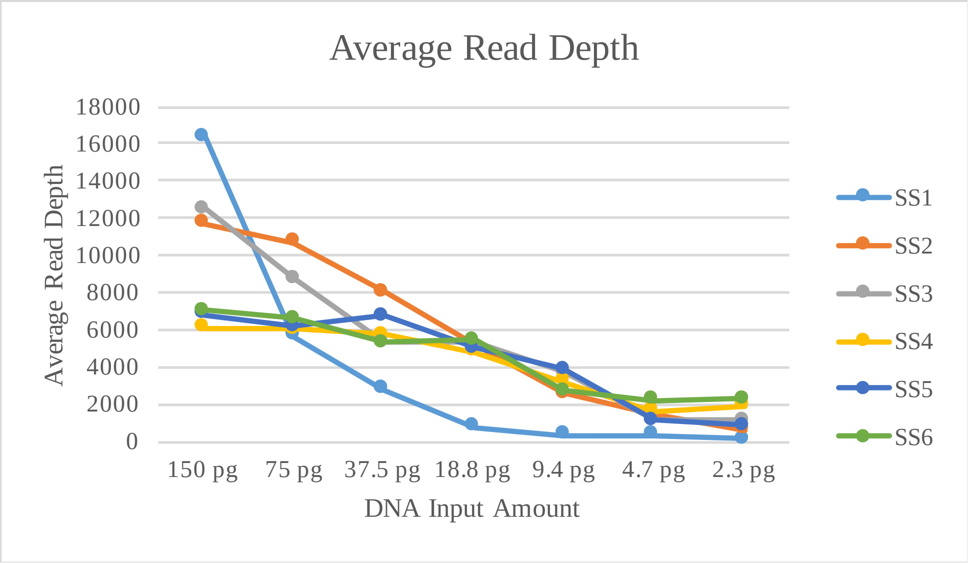

(B)

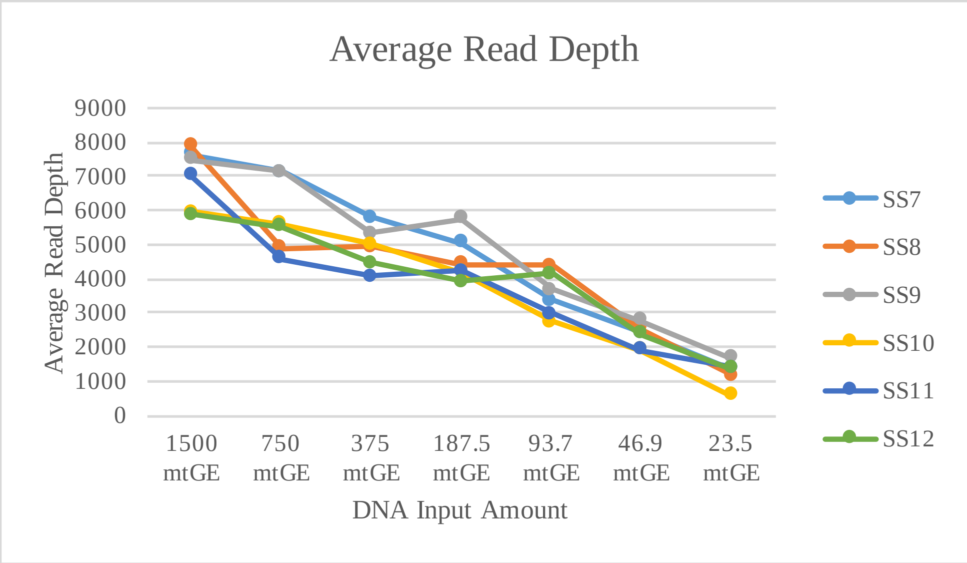

Figure S3. (A) Average read depth across the mtGenome for the six nuclear DNA quantitated dilution series samples. **(B)** Average read depth across the mtGenome for the six mtDNA quantitated dilution series samples.

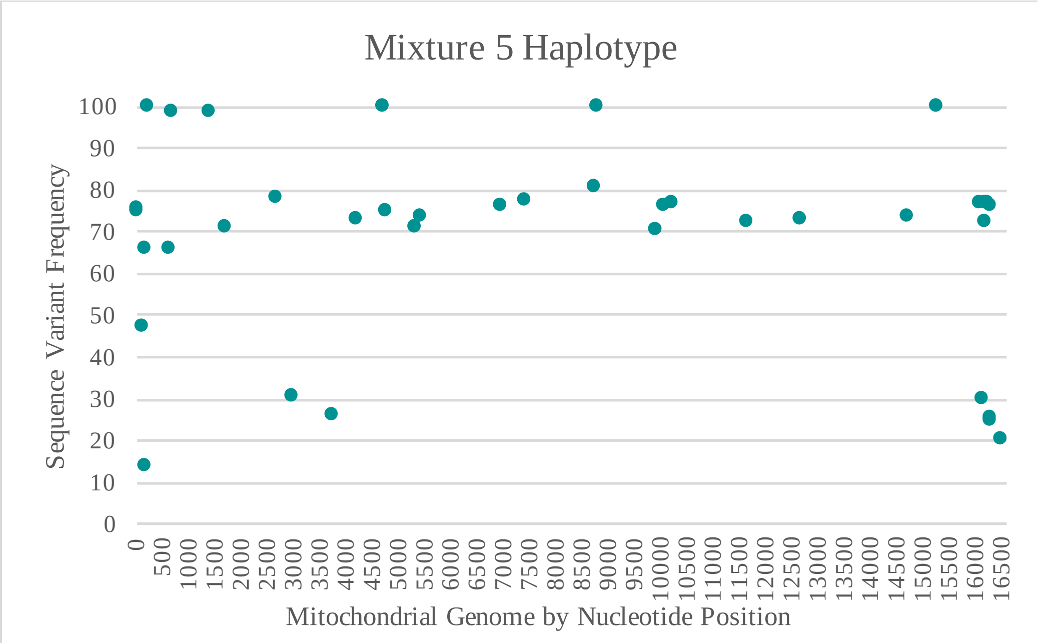

Figure S4. The sequence variant frequencies for one of the 2:1 mixtures included in Sequencing Run 31were plotted against the nucleotide position of that sequence variant to illustrate the quantitative contribution of each contributor to the total read depth of the mixed haplotype. The sequence variants present in both contributors, the major contributor, and the minor contributor cluster together with the personal point heteroplasmy present in each contributor falling out of these clusters.

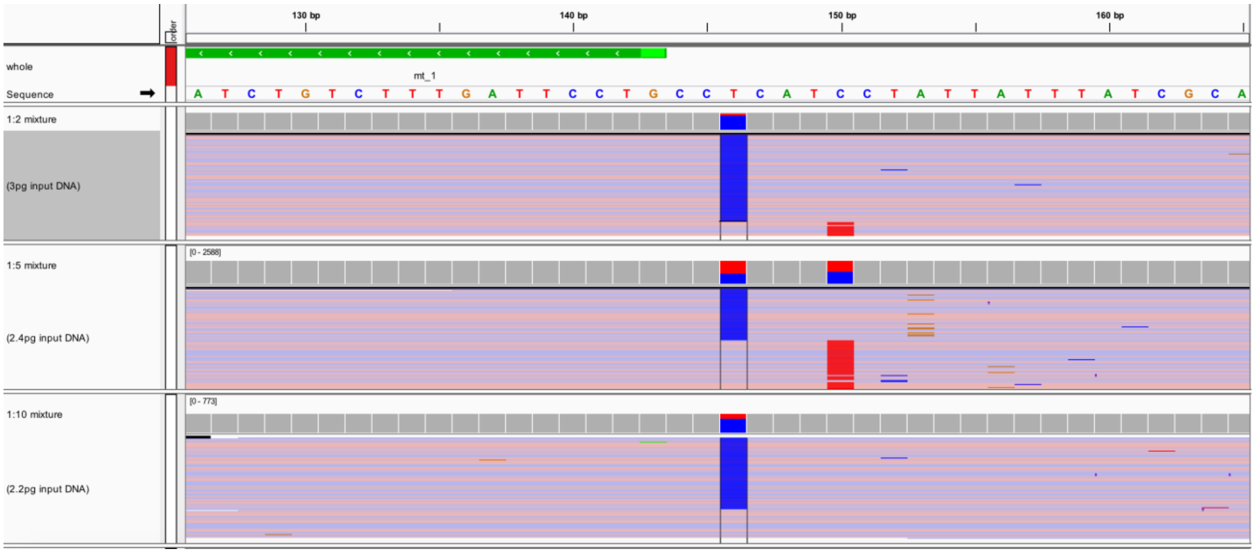

Figure S5. Screen shot from IGV illustrating the stochastic effects (i.e., variable sequence variant frequencies and drop-out) that can take place when amplifying lower amounts of input DNA, increasing the difficulty of deconvolution from these mixtures in mixture series 1 (Sequencing Run 29).

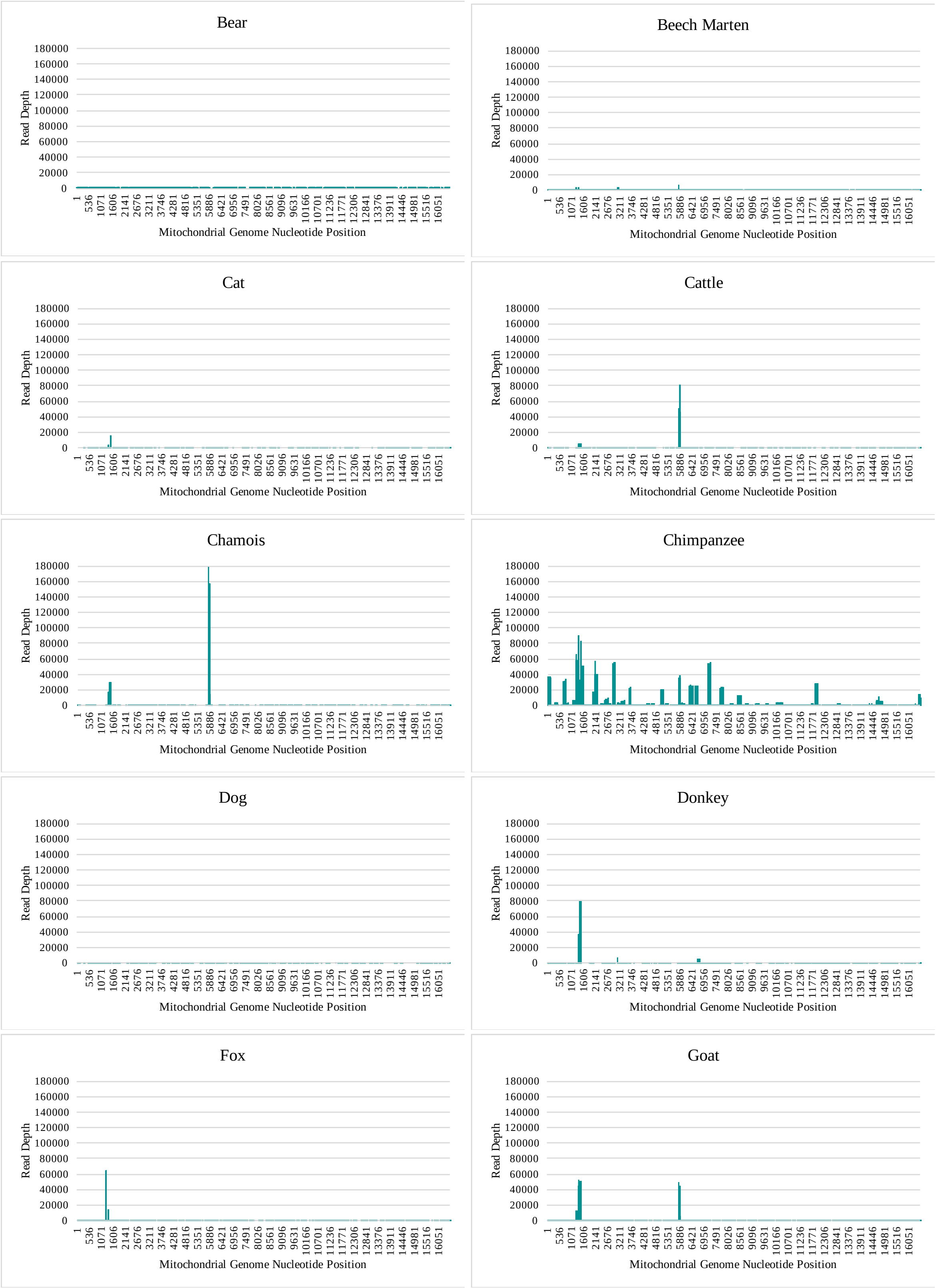
Bear
Read Depth
Mitochondrial Genome Nucleotide Position
Beech Marten
Read Depth
Mitochondrial Genome Nucleotide Position
Cat
Read Depth
Mitochondrial Genome Nucleotide Position
Cattle
Read Depth
Mitochondrial Genome Nucleotide Position
Chamois
Read Depth
Mitochondrial Genome Nucleotide Position
Chimpanzee
Read Depth
Mitochondrial Genome Nucleotide Position
Dog
Read Depth
Mitochondrial Genome Nucleotide Position
Donkey
Read Depth
Mitochondrial Genome Nucleotide Position
Fox
Read Depth
Mitochondrial Genome Nucleotide Position
Goat
Read Depth
Mitochondrial Genome Nucleotide Position

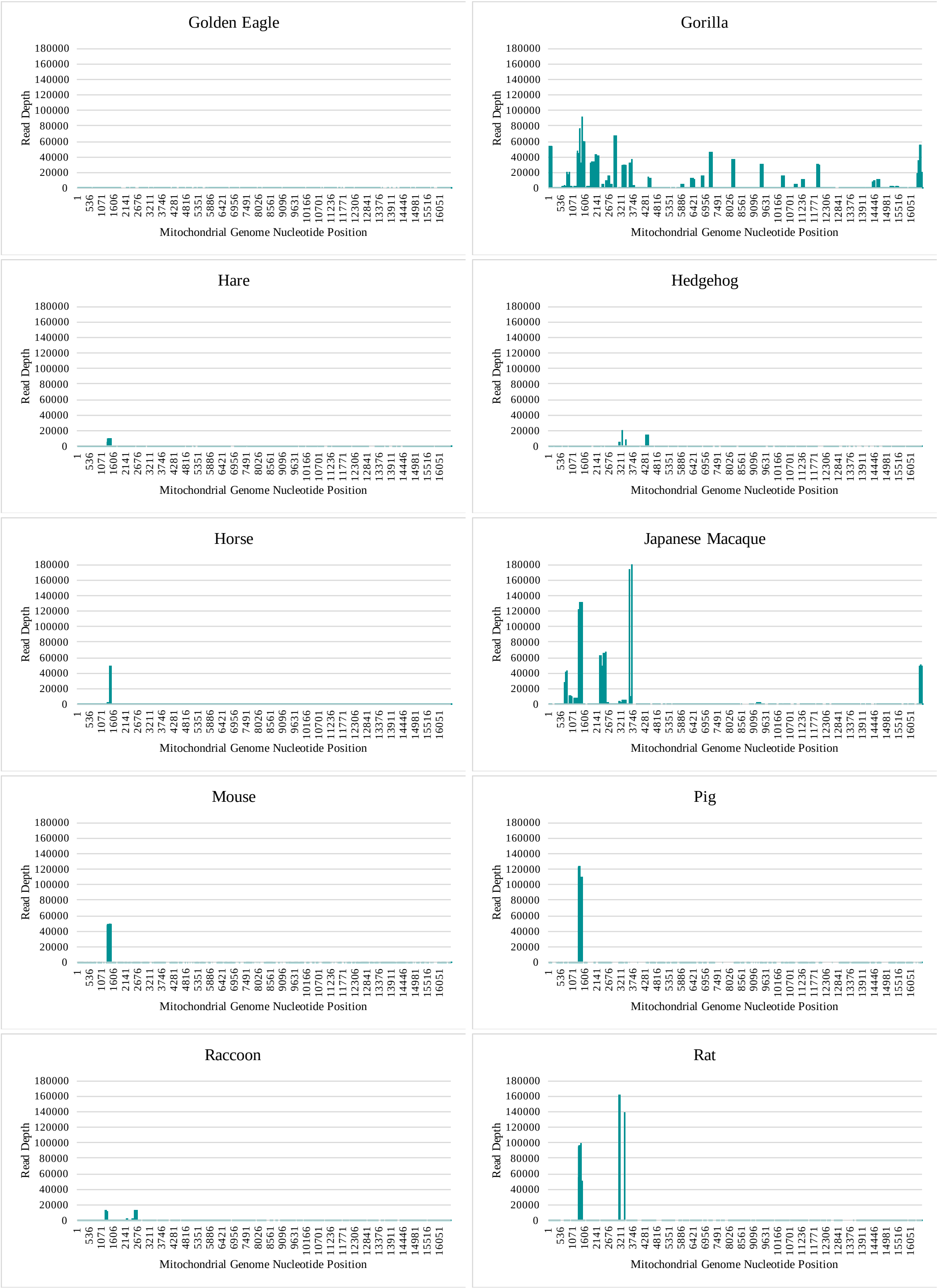
Golden Eagle
Gorilla
Hare
Hedgehog
Horse
Japanese Macaque
Mouse
Pig
Raccoon
Rat
Read Depth
Mitochondrial Genome Nucleotide Position
180000
160000
140000
120000
100000
80000
60000
40000
20000
0
1
536
1071
1606
2141
2676
3211
3746
4281
4816
5351
5886
6421
6956
7491
8026
8561
9096
9631
10166
10701
11236
11771
12306
12841
13376
13911
14446
14981
15516
16051

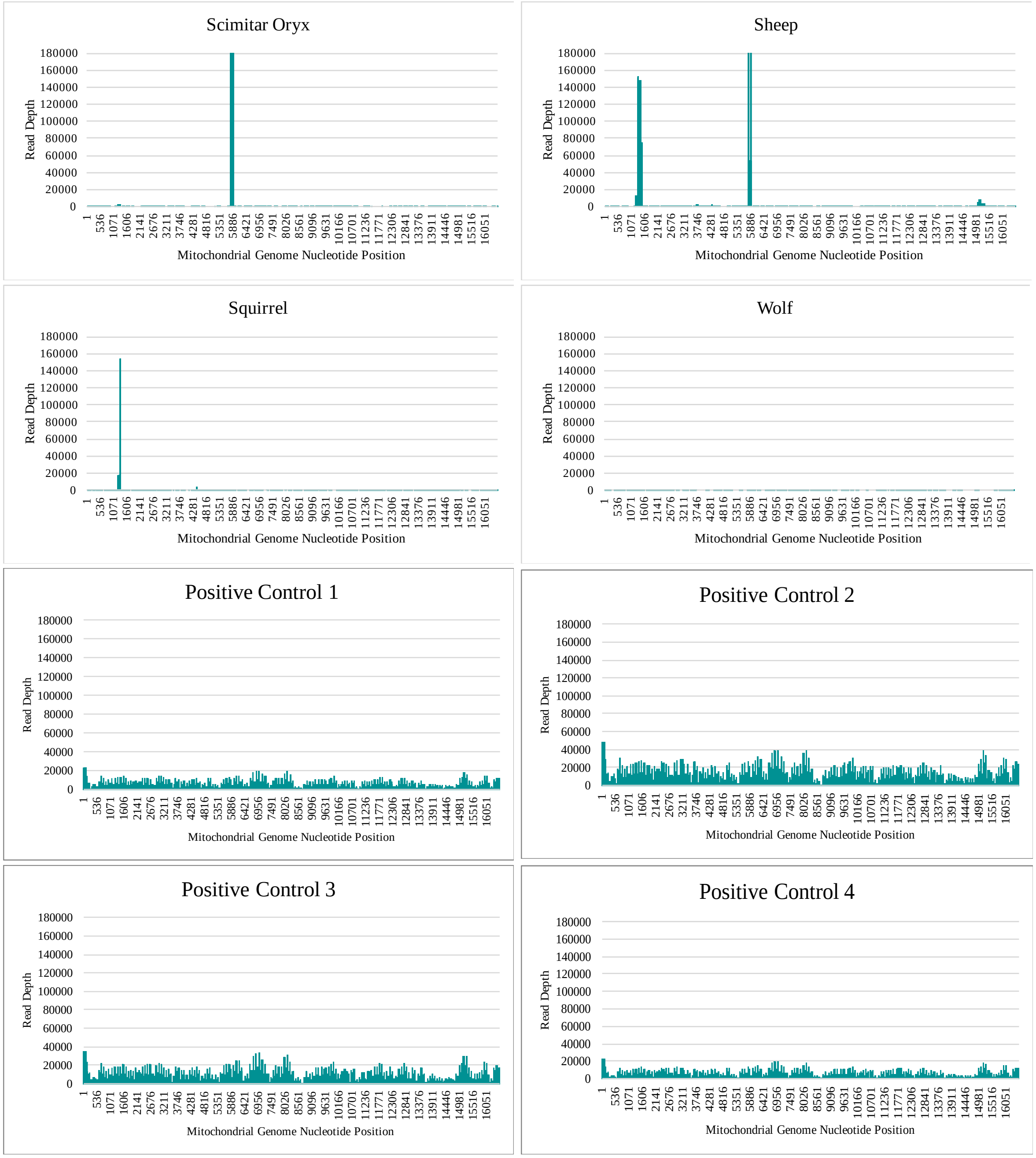
Scimitar Oryx
Sheep
Squirrel
Wolf
Positive Control 1
Positive Control 2
Positive Control 3
Positive Control 4
Read Depth
Mitochondrial Genome Nucleotide Position
0
20000
40000
60000
80000
100000
120000
140000
160000
180000
1
536
1071
1606
2141
2676
3211
3746
4281
4816
5351
5886
6421
6956
7491
8026
8561
9096
9631
10166
10701
11236
11771
12306
12841
13376
13911
14446
14981
15516
16051

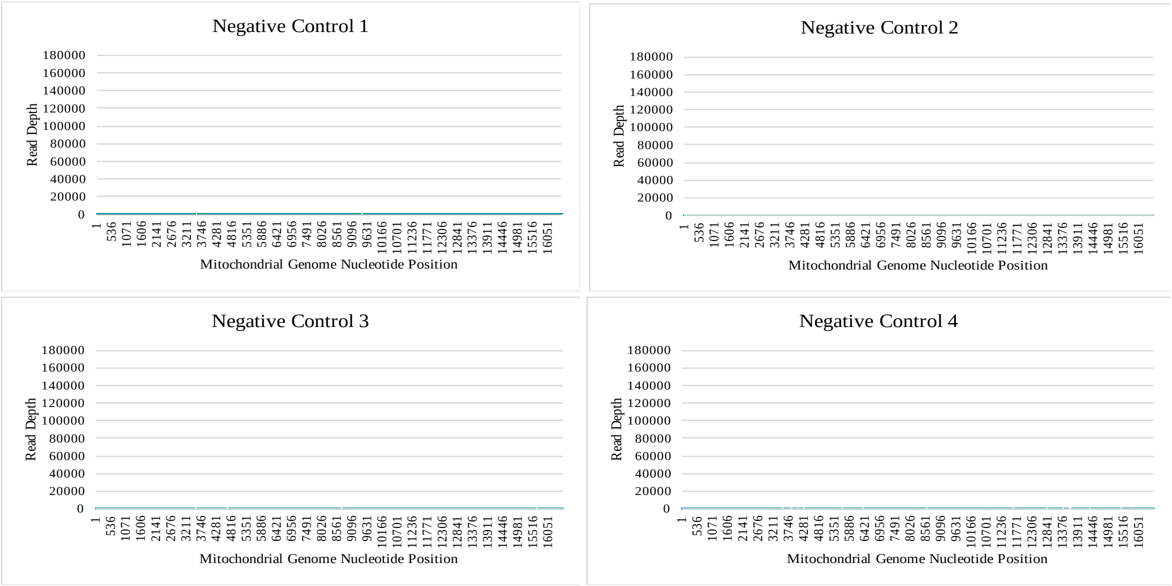

Figure S6. Read depth for each nucleotide position across the rCRS reference genome for each of the 24 vertebrate DNAs, four positive controls, and four negative controls included in the species specificity run. The X- and Y-axis for each chart has been scaled the same to allow for comparisons.
